# *Salvia guidongensis* sp. nov.: unraveling a critical evolutionary link in East Asian *Salvia* from Central China integrating morphology, phylogeny, and plastid genomics

**DOI:** 10.3389/fpls.2024.1332443

**Published:** 2024-03-05

**Authors:** Yan-Bo Huang, Zhe-Chen Qi, Jie-Ying Feng, Bin-Jie Ge, Cun-Zhong Huang, Yu-Qing Feng, Jing Wu, Pu-Rui Wei, Takuro Ito, Goro Kokubugata, Pan Li, Yu-Kun Wei

**Affiliations:** ^1^ Eastern China Conservation Centre for Wild Endangered Plant Resources, Shanghai Chenshan Botanical Garden, Shanghai, China; ^2^ Zhejiang Province Key Laboratory of Plant Secondary Metabolism and Regulation, College of Life Sciences and Medicine, Zhejiang Sci-Tech University, Hangzhou, China; ^3^ Natural Resources Bureau of Guidong County, Chenzhou, China; ^4^ Laboratory of Systematic & Evolutionary Botany and Biodiversity, College of Life Sciences, Zhejiang University, Hangzhou, China; ^5^ East China Survey and Planning Institute of the National Forestry and Grassland Administration, Hangzhou, China; ^6^ Tohoku University Botanical Gardens, 12-2 Kawauchi, Aoba-ku, Sendai-shi, Miyagi, Japan; ^7^ Department of Botany, National Museum of Nature and Science, Tsukuba, Ibaraki, Japan; ^8^ Shanghai Engineering Research Centre of Sustainable Plant Innovation, Shanghai Botanical Garden, Shanghai, China

**Keywords:** biogeography, East Asia, lamiaceae, plastome, phylogenetic analysis, Sino-Japanese flora, taxonomical link

## Abstract

**Introduction:**

*Salvia* L., representing the largest genus within the mint family, is noted for its global distribution of approximately 1000 species, with East Asia, and particularly China, recognized as a critical center of diversity for the genus.

**Methods:**

Our research was conducted through extensive fieldwork in Guidong County, Hunan Province, China, where we identified a previously undescribed species of *Salvia*. The identification process involved detailed morphological observations, phylogenetic analyses, and plastid genomics.

**Results:**

The newly discovered species, *Salvia guidongensis*, exhibits unique characteristics not commonly observed in the East Asian lineage of Salvia, including dual floral colors within natural populations—either pale purple or pale yellow. Morphologically, while it shares similarities with members of sect. *Glutinaria*, *S. guidongensis* is distinct in its floral morphology, stature, and specific foliar traits. Phylogenetic analysis places *S. guidongensis* in a unique clade within the East Asian lineage of *Salvia*, suggesting it may serve as an important evolutionary link. Additionally, we explored the plastome features of *S. guidongensis*, comparing them with those of closely related species.

**Discussion:**

The discovery of *S. guidongensis* not only entriches the taxonomic tapestry of *Salvia* but also provides critical insights into the biogeography and evolutionary pathways of the genus in East Asia. By integrating morphological and molecular data, we validate the novel status of *S. guidongensis* and highlight its significance in bridging taxonomic and evolutionary gaps within Sect. *Glutinaria* of *Salvia*.

## 1 Introduction

*Salvia* L., representing the largest genus within the Lamiaceae family, encompasses an estimated 1000 species. Its widespread distribution across both the Old and New Worlds, combined with its rich morphological diversity and unique pollination strategies, has consistently drawn the attention of botanists and ecologists (Drew et al., 2017; Hu et al., 2018; Kriebel et al., 2019; Drew, 2020; Kriebel et al., 2020). East Asia, in particular, stands out as significant hub of *Salvia* diversity, housing approximately 100 species. Remarkably, China alone is home to over 80 distinct species (Wu, 1977; Hu et al., 2018; Su et al., 2022). Recent years have witnessed the discovery of new species within China, further enriching the genus’s diversity (Hu et al., 2014; Wang et al., 2016; Xiang et al., 2016; Hu et al., 2017; Wei et al., 2019, 2021). The vast morphological diversity, spanning root to stamen, coupled with varied habitats, makes taxonomic studies of East Asian *Salvia* a formidable challenge (Wei et al., 2015; Su et al., 2022).

Recent research has predominantly placed East Asian *Salvia*, with the exception of *S. grandifolia* and *S. deserta*, into the newly established subg. *Glutinaria* (Hu et al., 2018, 2020). Within this subgenus, Hu et al. (2018) initially delineated eight distinct sections. Notably, the section *Glutinaria*, comprising six species, has been identified as a strongly supported monophyletic clade, occupying a relatively basal position within the East Asia *Salvia* lineage (Hu et al., 2018; Zhao et al., 2020; Su et al., 2022). Yet, the interspecific relationships within this clade warrant further exploration. The species within this clade exhibit a fascinating disjunct distribution, spanning from Europe and Central Asia (*S. glutinosa*) to the Himalayas (*S. nubicola*), and further extending to East China (*S. nipponica*), Korea (*S. chanryoenica*), and Japan (*S. nipponica*, *S. koyamae*, *S. glabrescens*). Morphologically, these species bridge the characteristics of subg. *Salvia* and subg. *Sclarea* according to traditional classification (Hu et al., 2018), showcasing features such as simple leaves, tubular-campanulate calyces, and falcate upper corolla lips.

In August 2021, during our fieldwork in Guidong County, eastern Hunan Province, central China, we encountered a unique *Salvia* population. Morphologically, this population aligns with sect. *Glutinaria*, a section previously undocumented in central China. While these individuals bear similarities to *S. nubicola* and *S. nipponica*, distinct differences in leaf morphology, flower color, and corolla structure are evident. Notably, this population exhibits flowers in both pale yellow and pale purple colors, a rarity in the East Asian lineage of *Salvia*. Subsequent field investigations in 2022 allowed us to collect specimens of both color variants. Our herbarium work did not yield any existing specimens corresponding to this new species. To ascertain its systematic position, we incorporated this new species, along with all extant species from sect. *Glutinaria* and representatives from six sections of subg. *Glutinaria*, into our phylogenetic analyses. The combined evidence from unique morphology and systematic positioning confirms the novelty of this species, which we describe and illustrate in the subsequent sections. Furthermore, we employed comparative plastid genomics to discern the genetic characteristic features within this section.

## 2 Materials and methods

### 2.1 Morphological observation

Between 2021 and 2022, we undertook multiple field trips to Qiyunfeng National Forest Park, located in Guidong County, Hunan Province, China, specifically to study this species. Specimens with flowers were collected in 2022. During our 2022 expedition, we collected specimens bearing flowers. Concurrently, we consulted several of China’s primary herbaria, including CSH, HUST, KUN, LBG, NAS, PE, acronyms as per Thiers (2020). Despite our extensive search, we did not identify any additional specimens corresponding to this new species in these herbaria. Our field observations and collected samples facilitated a comprehensive documentation of the morphology of this novel species. We systematically quantified morphological characters, presenting both quantitative and qualitative data for *S. guidongensis* and its phylogenetically proximate relatives within Sect. *Glutinaria* (*S. nubicola*, *S. glabrescens*, *S. chanryoenica*, *S. nipponica*, and *S. glutinosa*). Additionally, we analyzed the ecological feature of flowering time as a qualitative trait. This analysis covered a total of 155 individuals, with detailed trait information provided in **Supplementary Table S3**. A Principal Component Analysis (PCA) was conducted to analyze the variation in trait data among species. During data preparation, the qualitative traits were converted into dummy variables. For quantitative data, missing values were imputed using the mean value of each trait. Prior to PCA, the data were standardized. The entire data analysis and visualization process was performed using Python packages: Pandas for data transformation, Scikit-learn for standardization and PCA execution, and Matplotlib for visualization. To ensure. a robust and reliable PCA, we include only those individuals with complete or nearly complete datasets for both quantitative and qualitative traits (note that a few quantitative traits were not successfully measured for some individuals). This subset included 34 individuals of *S. guidongensis*, 20 of *S. nubicola*, 10 of *S. glabrescens*, 9 of *S. chanryoenica*, 11 of *S. nipponica*, and 10 of *S. glutinosa*. Following the PCA, we utilized statistical approach to account for the nested structure of our data. Specifically, we adopted Mixed Effects Models for each quantitative trait, incorporating ‘species’ as a fixed effect and ‘population’ as a random effect. This approach acknowledges the potential non-independence of observations within the same population. Following the mixed model analysis, we applied Tukey’s Honest Significant Difference (HSD) test for *post hoc* comparisons between species. We set a significance threshold at a *p*-value of < 0.05. The outcomes of these analyses were effectively visualized using violin plots.

### 2.2 Molecular methods, phylogenetic analyses, and divergence time estimation

For our phylogenetic investigations, we included samples from both red and yellow-flowered individuals of the newly discovered species. Additionally, our sample set encompassed twelve *Salvia* species, which comprised all existing species from sect. *Glutinaria* and representative species from six sections of subg. *Glutinaria*. Based on prior phylogenetic research, *S. petrophila* was designated as the outgroup. Voucher specimens for all sampled species have been deposited at the Herbarium of Chenshan Botanical Garden (CSH), as detailed in **Supplementary Table S1**.

Genomic DNA extraction was performed on silica-dried tissue using a modified cetyltrimethylammonium bromide (CTAB) protocol (Li et al., 2013). The DNA, post chloroform/isoamyl alcohol extraction from the aqueous phase, was precipitated using isopropanol and subsequently resuspended in Tris-ethylenediamine tetra-acetic acid (TE) buffer (pH 8.0). For the purpose of addressing inter-specific phylogenetic relationships, we selected eight plastid DNA fragments (*mat*K, *ndh*A, *ndh*F, *psb*A-*trn*H, *rbc*L, *rpl*16, *trn*L-F, and *ycf*1-*rps*15). The amplification procedures for *psb*A-*trn*H, *rbc*L, *trn*L-F, and *ycf*1-*rps*15 were adopted from Hu et al. (2018). The *ndh*A fragment was amplified using primers designed as per Shaw et al. (2007). For the *mat*K fragment, primer design and amplification followed the methodology of Bendiksby et al. (2011). The primer design and amplification protocols for *ndh*F and *rpl*16 followed Scarcelli et al. (2011). Detailed information on the primers used in this study are provided in **Supplementary Table S2**. The PCR cycling conditions were template denaturation at 94°C for 6 min prior to the start of PCR cycles, then amplified for 35 cycles of 1 min at 94°C, 1.5 min at 53°C, 2 min at 72°C, concluding with a 12-min extension at 72°C. The PCR products were sent to Qingke Ltd., Hangzhou, China, for Sanger sequencing. Sequences from both forward and reverse directions were assembled using GENEIOUS v11.1.5 (Biomatters Ltd., Auckland, New Zealand). All sequences have been deposited in GenBank, as detailed in **Supplementary Table S1**.

Phylogenetic relationships were inferred using both Bayesian inference (BI) and maximum likelihood (ML) methodologies. For the ML analysis, we employed IQTREE v1.6.8 (Nguyen et al., 2015), utilizing the best-fit model of sequence evolution as determined by ModelFinder (Kalyaanamoorthy et al., 2017) which is included in the software package. The model selection was based on the Bayesian Information Criterion. The bootstrap support (BS) values were derived from 5,000 replicates, employing the best-fit TPM3u+F+R2 model. For the BI analyses and divergence time estimation, we employed BEAST v2.4.3 (Bouckaert et al., 2014) using an uncorrelated lognormal relaxed-clock model (Bouckaert et al., 2014). A birth-death model of tree priors was implemented (Drummond and Bouckaert, 2015). Because of no reliable fossil records in *Salvia*, we adopted an estimated crown time of subg. *Glutinaria* (12.57–23.87 Ma, Kriebel et al., 2019) as a calibration at the root. Two independent runs, each spanning 100 million generations, were conducted, saving samples at intervals of every 5,000 generations. Convergence and burn-in were assessed using Tracer v1.7 (Rambaut et al., 2018), leading to the discarding of the initial 10% of trees. The log files were checked for convergence using Tracer. Convergence was further confirmed by ensuring all ESS (explained sum of squares) values exceeded 200 in the log files. A maximum clade credibility tree, featuring median branch lengths and annotated with posterior probability (PP) values and 95% highest posterior density (HPD) intervals at nodes, was generated and summarized using TreeAnnotator v1.8.4, part of the BEAST package. The resultant trees were visualized and interpreted using FigTree v1.4.3 (Rambaut, 2021).

### 2.3 Chloroplast genomic analyses and comparative insights in *S. guidongensis* and closely related *Salvia* species

#### 2.3.1 Plastome sequencing, assembly, and annotation

For our comparative analyses, we focused on the complete plastomes of four *Salvia* species: the newly described *S. guidongensis*, *S. nubicola*, *S. glutinosa*, and *S. chanryoenica*. Among these, the genomes of *S. guidongensis* and *S. nubicola* were sequenced in this study, while the plastomes of *S. glutinosa* and *S. chanryoenica* were sourced from GenBank (accessions: NC067736 and MH261357, respectively). Sequencing were executed on the Illumina HiSeq-2500 platform (Illumina Inc., San Diego, CA, USA), yielding an average of approximately 50 million high-quality, adaptor-trimmed reads (150 bp pair-end read length) for each species. Sequence alignment, assembly, and annotation were facilitated by tools including GetOrganelle v1.7.0c (Jin et al., 2020), MAFFT (Katoh and Standley, 2013), GeSeq (Tillich et al., 2017) and GENEIOUS v11.0.5. The Organellar Genome DRAW tool was employed to visualize the circular structure of plastomes (Lohse et al., 2013). We further compared and analyzed boundary regions between the LSC, IR and SSC using the respective plastomes. Sequence identity across the four *Salvia* plastomes was assessed using the online software mVISTA (http://genome.lbl.gov/vista/mvista/submit.shtml) (accessed on 22 February 2023) with the Shuffle-LAGAN mode (Frazer et al., 2004).

#### 2.3.2 Repeat sequence and SSR analysis

The position and size of three repeat sequence types, including direct (forward), inverted (palindromic) and reverse repeats, were identified in the four *Salvia* plastomes using the online REPuter program (Kurtz et al., 2001). The constraints set for these repeat types were: a minimum repeat size of 30 bp and a sequence identity exceeding 80%, with a hamming distance of 3 (i.e., the gap size between repeats larger than 3 bp). Simple sequence repeats (SSRs) were detected using the MIcroSAtellite (MISA) perl script (Thiel et al., 2003) with a threshold for mono-, di-, tri-, tetra-, penta-, and hexanucleotide SSRs containing 10, 6, 5, 3, 3, and 3 repeat units, respectively.

#### 2.3.3 Codon usage analysis

We employed CodonW 1.4.2 (available at http://codonw.sourceforge.net/) to analyze the Relative Synonymous Codon Usage (RSCU) and the Effective Number of Codons (ENC) for 62 protein-coding sequences (CDS) from four *Salvia* plastomes. Additionally, the Codon Adaptation Index (CAI) was determined. CAI values range between 0 and 1, with higher values indicating stronger codon bias. Subsequently, the online analysis tool CUSP (accessible at http://imed.med.ucm.es/EMBOSS/) was utilized to compute the overall GC content (GC all) for each species’ CDS. This tool also provided the GC content at the first, second, and third positions of codons, denoted as GC1, GC2, and GC3, respectively. Using GC3 as the abscissa and ENC as the ordinate, an ENC plot was constructed based on the standard curve calculation formula, as described by He et al. (2019).

## 3 Results

### 3.1 Morphological comparison

**Figure 1:**
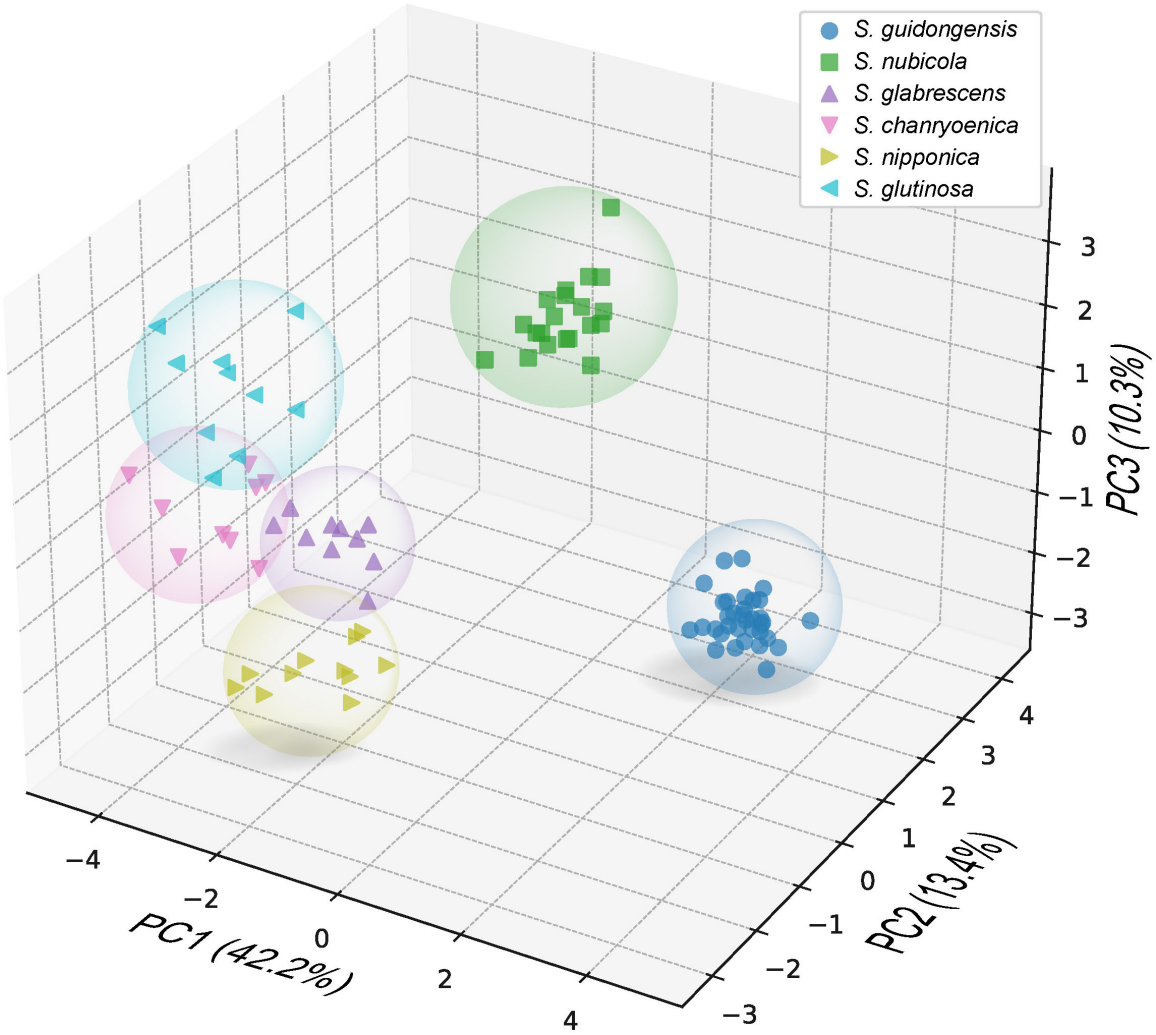
Principal Component Analysis (PCA) using 26 morphological characters of *S. guidongensis* and five closely related species within sect. *Glutinaria*. Dots in the plots represent analyzed individuals of species. The first principal component (PC1) accounts for 42.2% of the total variance, followed by PC2 with 12.8%, and PC3 with 10.3%.

**Figure 2:**
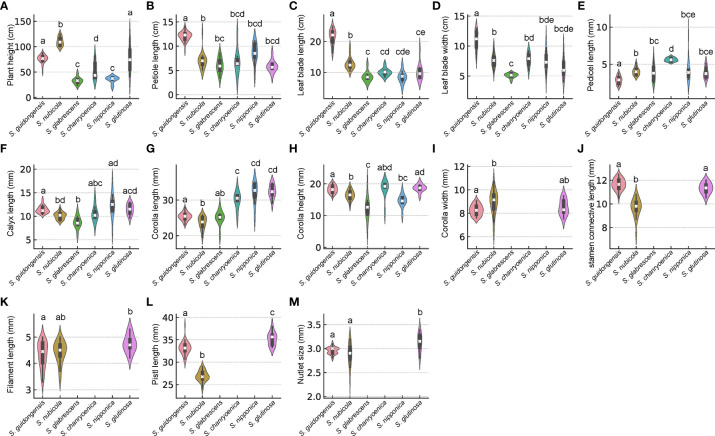
Violin plots depicting the distribution of comparisons of morphological characteristics among six *Salvia* species in sect. *Glutinaria*. White cross indicates the median, the box indicates the upper and lower quartiles. The thin line represents the ‘whiskers’, which extend to 1.5 times the interquartile range from the upper and lower quartiles. Different small letters indicate significant differences within morphological traits among species (p <0.05). **(A)** Plant height; **(B)** Petiole length; **(C)** Leaf blade length; **(D)** Leaf blade width; **(E)** Pedicel length; **(F)** Calyx length; **(G)** Corolla length; **(H)** Corolla height; **(I)** Corolla width; **(J)** stamen connective length; **(K)** Filament length; **(L)** Pistl length; **(M)** Nutlet size.

*Salvia guidongensis* shows typical synapomorphies of sect. *Glutinaria*, including simple leaves, tubular-campanulate calyces, falcate upper corolla lips, unequal connective arms, and fused deformed posterior thecae. However, it stands out prominently among its congeners by a unique combination of morphological attributes. The Principal Component Analysis (PCA) results indicate that PC1, PC2, and PC3 account for 42.2%, 13.4%, and 10.3% of the variance, respectively, totaling 65.9%. In the PCA plot, *S. guidongensis* is positioned distinctly (**Figure 1**), with leaf blade length and corolla upper & lower lip type as primary contributors to PC1, corolla length, pistil length, and stamen connective length to PC2, and flowering time, plant height, and corolla height to PC3. These findings corroborate our observations that the corolla of *S. guidongensis*, characterized by a narrower angle between the upper and lower lips, is distinct from other species within sect. *Glutinaria*. The pronounced inward curl of its middle lobe further underscores its uniqueness. In terms of floral coloration, *S. guidongensis* is exceptional, with natural populations showcasing either pale purple or pale yellow flowers, a rarity within subg. *Glutinaria*. Its multi-branched upright stem also differentiates it from the predominantly unbranched stems of *S. glabrescens*, *S. chanryoenica*, and *S. nipponica*. In the ANOVA of eleven quantitative traits, *Salvia guidongensis* exhibited significant differences in five traits compared to all other five species within the group, including petiole length, leaf blade length and width, pedicel length, and pistil length (**Figure 2**). Additionally, two traits, plant height and corolla length, showed significant differences when compared to four species within the group. Morphometrically, *S. guidongensis* reaches an intermediate height of 50–88 cm, positioning itself between the taller *S. nubicola* and *S. glutinosa* and the smaller species like *S. glabrescens* and *S. nipponica*. The ovate, elliptic leaves of *S. guidongensis* are among the largest in the group, and its leaf indumentum, being sparsely villous or glabrous, aligns more with *S. nubicola.* The distribution of character states for each analyzed qualitative trait, comprehensive morphological comparisons between *S. guidongensis* and its closely related species in sect. *Glutinaria*, and the coding methodology for qualitative morphological characters are detailed in **Supplementary Figure S1**, **Supplementary Tables S3**, and **S4**, respectively.

### 3.2 Molecular phylogenetic analyses and speciation time

**Figure 3:**
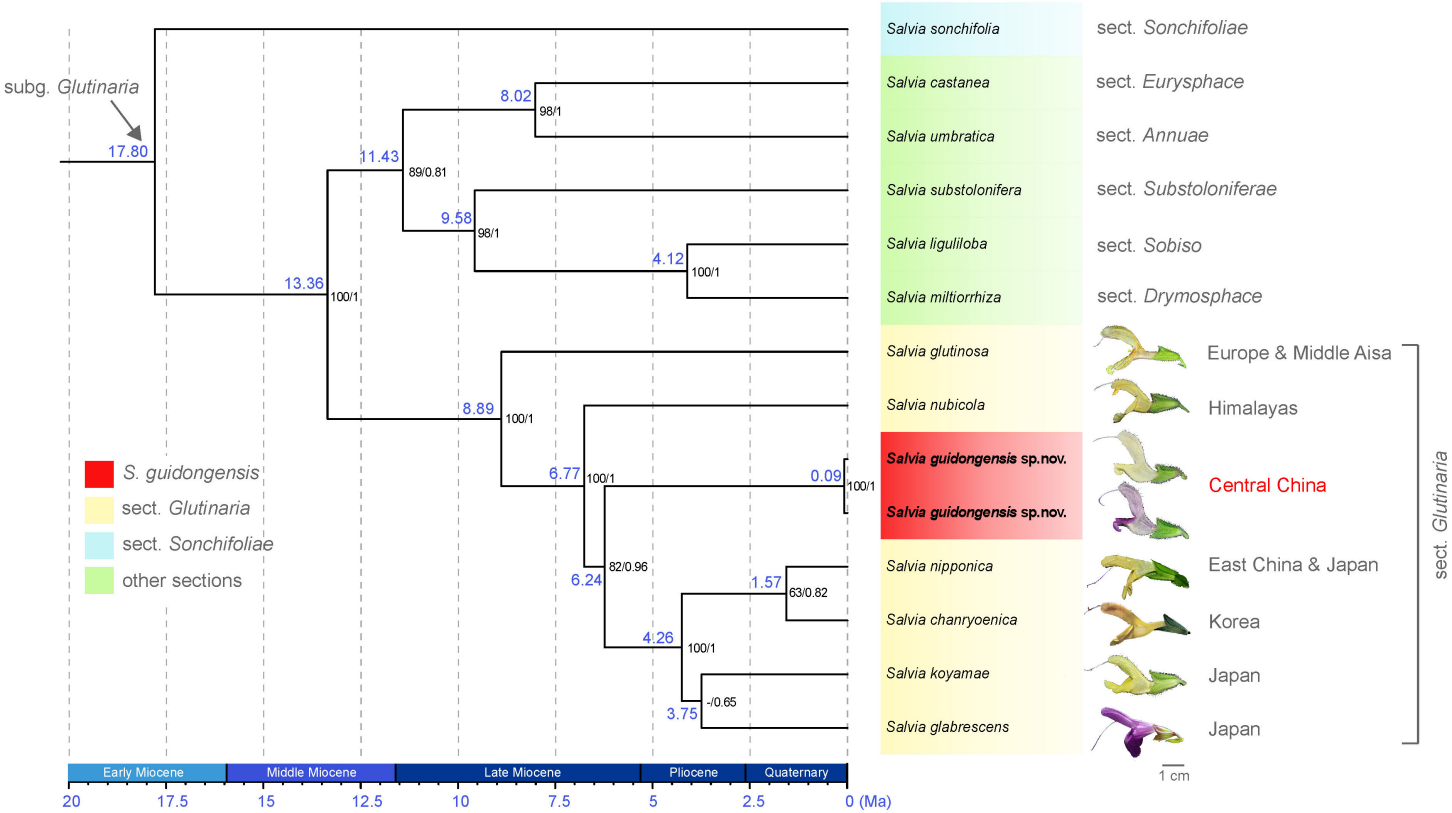
BEAST chronogram of *S. guidongensis* alongside 12 other *Salvia* species, illustrating a clade within subg. *Glutinaria* clade. Median node ages (in millions of years ago, Ma) are indicated on each branch. Bootstrap values from maximum likelihood (ML) analysis followed by posterior probabilities from Bayesian inference are indicated on each node. Representative flowers from species within sect. *Glutinaria* are depicted adjacent to their respective species name. The native distribution of each species was indicated. The timeline at the bottom provides a geological time scale measured in Ma.

The analysis of eight plastid (pt) regions—*mat*K, *ndh*A, *ndh*F, *psb*A-*trn*H, *rbc*L, *rpl*16, *trn*L-F, and *ycf*1-*rps*15—yielded an alignment of 17,028 bp, encompassing 706 variable sites, 230 of which were phylogenetically informative. Both ML and BI analyses produced congruent topologies; therefore, only the BEAST chronogram is presented (**Figure 3**; **Supplementary Figure S2**). The analyses robustly support the monophyly of species within sect. *Glutinaria* (ML BS = 100, BI PP = 1), indicating divergence within the group during the middle Late Miocene (ca. 8.89 Ma, **Figure 3**). Sect. *Glutinaria* is distinctively positioned as sister to all other sections in subg. *Glutinaria*, with the exception of the basal *S. sonchifolia* (sect. *Sonchifoliae*), dating back to approximately 17.80 Ma during Early Miocene. Within sect. *Glutinaria*, the early-diverging *S. glutinosa*, native to Europe and Middle Asia (ML BS = 100, BI PP = 1), is followed by the subsequent divergence of *S. nubicola* from the Himalayan region (ca. 6.77 Ma, ML BS = 100, BI PP = 1). This latter species is sister to the remaining taxa in the section. Intriguingly, *S. guidongensis*, originating in the late Late Miocene (ca. 6.24 Ma), forms a distinct clade from central China, sister to a monophyletic group of four species (ML BS = 82, BI PP = 0.96). This group, with a crown age in Pliocene (ca. 4.26 Ma), includes *S. nipponica* endemic to East China and Japan, *S. chanryoenica* from Korea, which diverged in the Quaternary (ca. 1.57 Ma), and a clade of two Japanese species, S. *koyamae* and *S. glabrescens*, though without pronounced support.

Given its distinctive phylogenetic placement, divergence time, morphological features, and singular geographical distribution, *S. guidongensis* emerges unequivocally as a novel species within *Salvia*.

### 3.3 Taxonomic treatment of the new species

**Figure 4:**
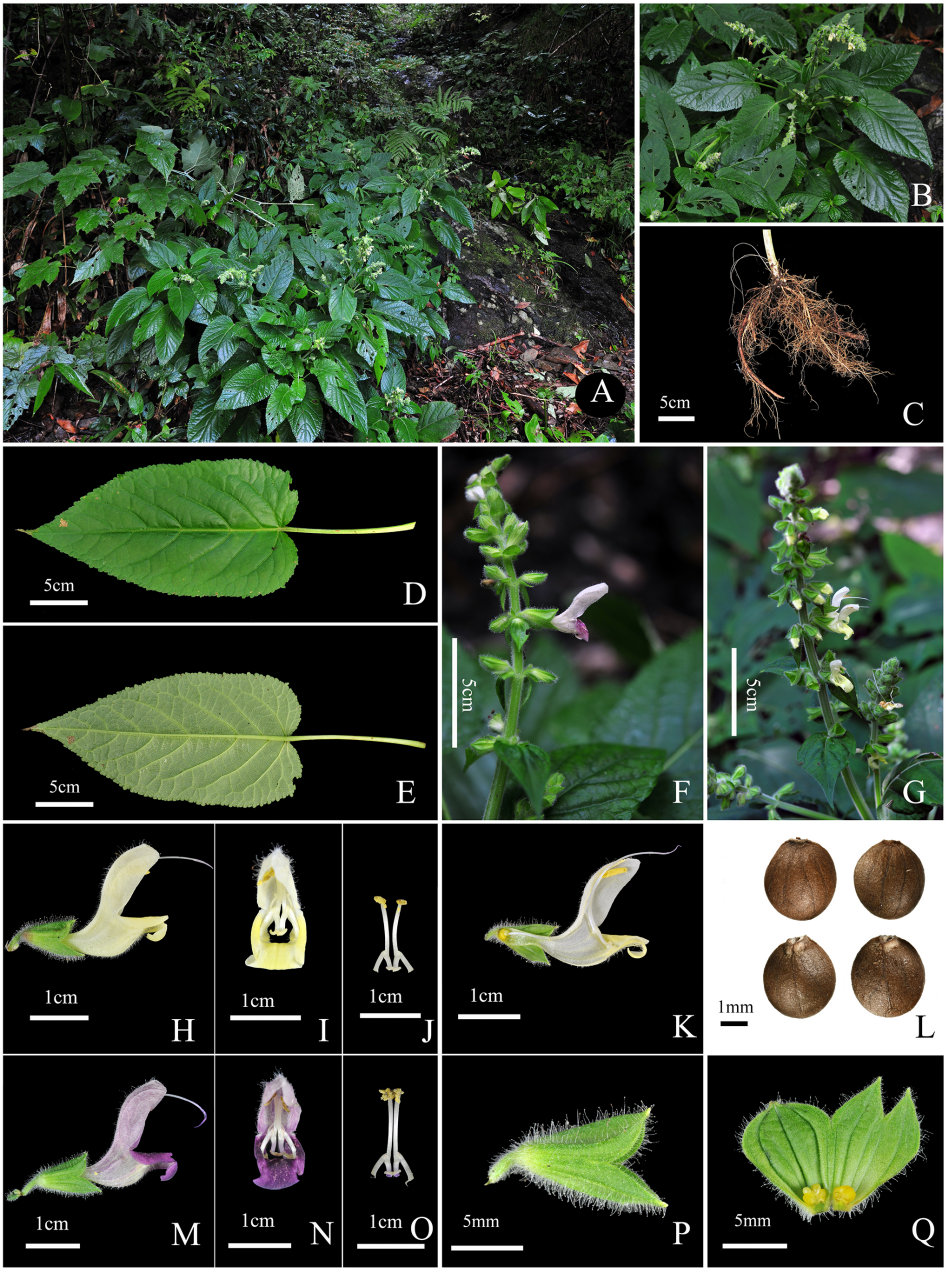
*Salvia guidongensis* C.Z. Huang, Yan.B. Huang, B.J. Ge, & Z.C. Qi, sp. nov. **(A)** Habitat (forest edge, beneath the forest near stream valleys); **(B)** Flowering plant; **(C)** Root; **(D)** Leaf adaxial (upper) surface; **(E)** Leaf abaxial (lower) surface; **(F)** Inflorescence (pale purple flowers); **(G)** Inflorescence (pale yellow flowers); **(H)** Corolla lateral view; **(I)** Corolla frontal view; **(J)** Stamens frontal view; **(K)** Corolla lateral view (longitudinal section); **(L)** Nutlets; **(M)** Corolla lateral view; **(N)** Corolla frontal view; **(O)** Stamens frontal view; **(P)** Calyx lateral view; **(Q)** Calyx inner surface spread out.

Taxonomic description of *Salvia guidongensis* C.Z. Huang, Yan.B. Huang, B.J. Ge, & Z.C. Qi, sp. *nov*. (**Figure 4**).

Type: CHINA. Hunan Province: Guidong County, Ou’jiang Town, Qiyunfeng National Forest Park, understory of subtropical broad-leaved forests, 1423 m, 113°58′43.01” E, 26°8′14.59” N, 24 August, 2022, Fl., *Yanbo Huang*, *Binjie Ge* & *Zhechen Qi* S1799 (holotype: CSH! Barcode number 0193180; isotypes: PE! Barcode number 0193179, KUN! Barcode number 0193178; paratypes: CSH! Barcode number 0193175–0193177, 0193181–85).

#### 3.3.1 Diagnosis

*Salvia guidongensis* is distinguished from other species in sect. *Glutinaria* by a unique combination of morphological traits:

-Leaves: Ovate to elliptic, markedly longer in both blade and petiole compared to congeners, exhibiting a dark green or yellow-green coloration. Leaf indumentum varies from sparsely villous to glabrous, similar to *S. nubicola*.

-Floral structures: The corolla is characterized by a narrower angle between the upper and lower lips. The middle lobe of the corolla exhibits a pronounced inward curl. Corolla length is relatively short, comparable to *S. nubicola* and *S. glabrescens*, with pedicel length typically shorter than in other species of the section.

-Floral coloration: Exhibits a rare dual floral coloration within natural populations, alternating between pale purple and pale yellow, a distinctive feature within sect. *Glutinaria*.

-Plant habit: Characterized by a multi-branched upright stem, in contrast to the predominantly unbranched stems of *S. glabrescens*, *S. chanryoenica*, and *S. nipponica*. The plant ranges from 50–88 cm, placing it between the taller *S. nubicola* and *S. glutinosa* and the shorter species like *S. glabrescens* and *S. nipponica*.

#### 3.3.2 Description

A perennial herb, standing erect. Stems upright and simple, ranging from multi-branched to unbranched, reaching a height of 50.0–88.0 cm. Petioles 9.0–14.0 cm in length; leaf blades ovate to elliptic, 16.0–25.0 × 9.0–13.0 cm, dark green to yellow-green, sparsely hispidulous or glabrous, apex acuminated, base cordate to rounded. Inflorescence densely villous, glandular pilose; verticillasters 6-flowered, in racemes or panicles; bracts sessile, ovate, shorter than calyx, abaxially fine pubescent, glandular hairy, margin long glandular hairy, apex acuminate. Pedicels 2.0–3.8 mm in length. Calyx 10.4–13.5 mm in length, densely glandular hairy. Corolla exhibits a unique pale purple to pale yellow coloration, 23.1–27.9 mm in length, 15.9–20.4 mm in height, and 7.6–9.1 mm in width; upper lip falcate, sparsely glandular hairy; lower lip’s middle lobe sharply inward curl. Filaments 3.3–5.0 mm; connectives 10.5–12.5 mm. Pistil extends 30.1–38.3 mm. Nutlets oblong to approximately globular, 2.8–3.1 mm × 2.3–2.8 mm. Flowering from August to October. Fruiting from September to November.

#### 3.3.3 Etymology

The species is named after the location of its first discovery, Guidong County, where it is currently exclusively distributed, making it an endemic species to this region. The specific epithet “*guidongensis*” is derived from “Guidong”, emphasizing its unique geographical association. Due to its morphological resemblance to *S. nubicola*, known in Chinese as ‘云生丹参’ (Yunsheng Danshen), we propose the Chinese name for *S. guidongensis* as ‘桂东丹参’ (Guidong Danshen).

#### 3.3.4 Additional specimens examined (Paratypes)

CHINA. Hunan Province: Guidong County, Ou’jiang Town, Qiyunfeng National Forest Park, 24 August, 2022, Fl., *Yanbo Huang*, *Binjie Ge*, *& Zhechen Qi S1800* (CSH); same locality, 27 August, 2019, Fl. *Cunzhong Huang, HCZ00203* (CSH).

#### 3.3.5 Distribution and habitat

*Salvia guidongensis* is uniquely found in Qiyunfeng National Forest Park in Ou’jiang Town, Guidong County, Hunan Province, China. This park, spanning 12,078 hectares, is characterized by a subtropical eastern humid monsoon climate and a rich vertically diversity of vegetation. It boasts a high vegetation cover rate of 92.47%, hosting a wide range of vascular plants across 209 families, 798 genera, and a staggering 1,577 species (Zhu et al., 2019). However, *S. guidongensis* is confined to a specific area within the park, thriving on limestone slopes near stream gullies, nestled in the understory of subtropical broad-leaved forests at an elevation of approximately 1400 meters.

The habitat of *S. guidongensis* is marked by a diversity flora, including key accompanying species like *Siphocranion nudipes* (Hemsl.) Kudô, *Polygonum chinense* L., and various species of *Lindera* Thunb., *Chamabainia* Wight, and *Impatiens* L. These plants typically inhabit moist, shaded environments (Čuda et al., 2014; Chen et al., 2019). Additionally, the presence of *Pilea* Lindl., *Oreocharis* Benth., and *Matsumurella kwangtungense* (C.Y.Wu) Bendiksby suggests an affinity for nutrient-rich, well-drained soils, characteristic of limestone terrains. The area also features *Clematis crassifolia* Benth., *Dioscorea japonica* Thunb., *Hylodesmum oldhamii* (Oliv.) Ohashi & Mill, *Hylodesmum podocarpum* (DC.) Ohashi & Mill, and *Yinshania* Ma & Zhao, along with understory ferns such as *Arachniodes aristata* (Forst.) Tindale and *Pteridium latiusculum* (Desv.) Hieron, all of which are indicative of a well-established, mature forest ecosystem (Qi et al., 2005; Marrs and Watt, 2006). The ecological tapestry is further enhanced by species such as *Lecanthus peduncularis* (Royle) Wedd., *Begonia pedatifida* H.Lév., *Cladrastis* Raf., and *Eomecon chionantha* Hance, along with others such as *Manglietia fordiana* Oliv., *Angelica biserrata* (Shan & Yuan) Yuan & Shan, and *Carex scaposa* Clarke, highlighting the area’s ecological complexity and biodiversity. This indicates that *S. guidongensis* an integral member of a unique ecological community, adapting to the forests dominated by relict evergreen broad-leaved species in eastern Hunan province, central China (Tang et al., 2018).

The limited distribution of *S. guidongensis* to this specific location, coupled with its small population size of 50–60 individuals, underscores its heightened vulnerability. Despite extensive searches in similar habitats in the vicinity, no further individuals have been discovered. Given its restricted range and diminutive population, *S. guidongensis* could potentially be classified as Critically Endangered (CR) under IUCN criteria B and C (IUCN, 2022). It is imperative to undertake additional field studies to amass a more holistic understanding of its distribution, population trends, potential threats, and other pertinent factors, which would aid in refining its conservation assessment.

### 3.4 Comparative plastomes of *S. guidongensis* and its relatives

#### 3.4.1 Genome organization and features

**Figure 5:**
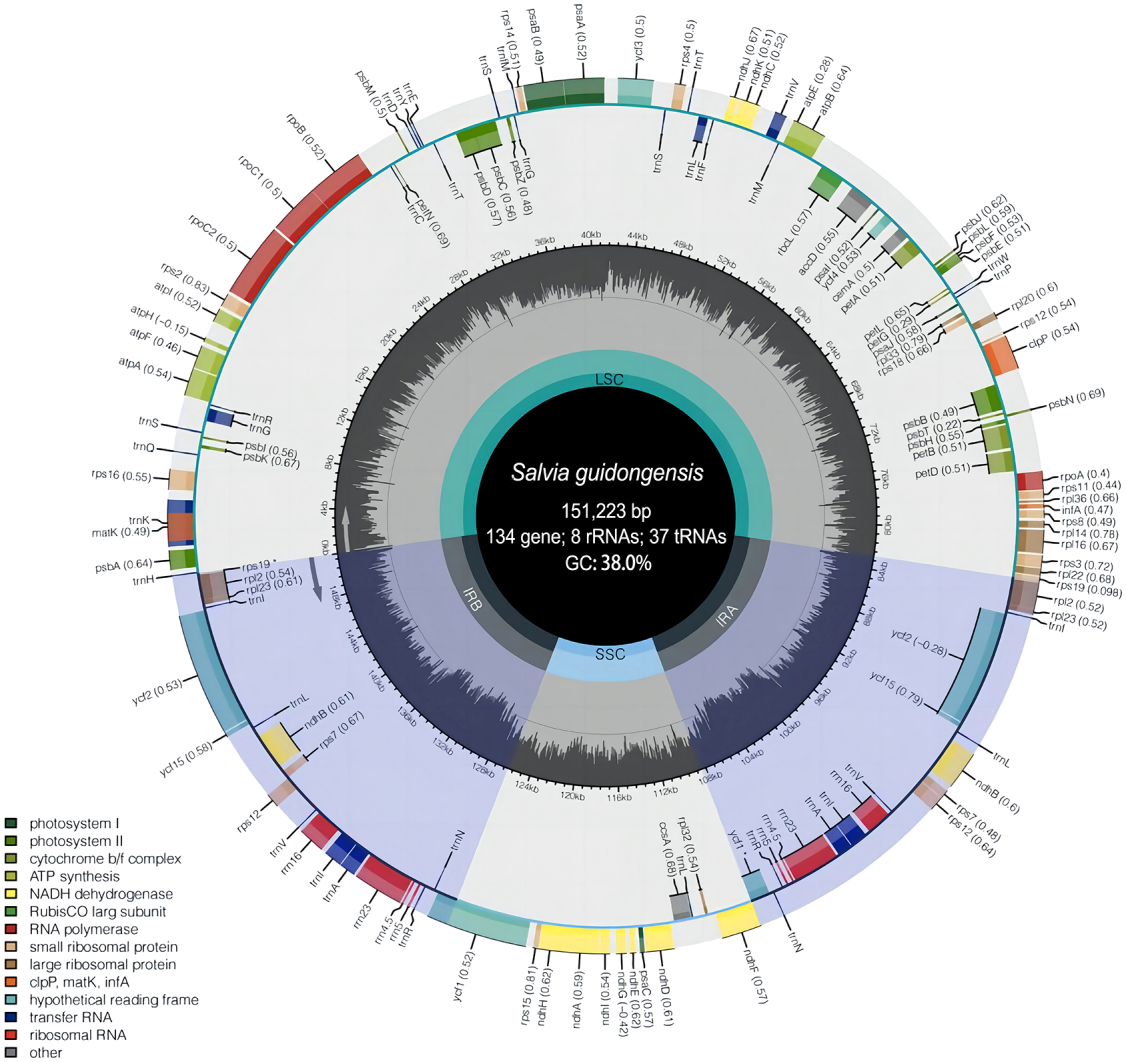
Plastome map of *S. guidongensis*. The inner dark gray circle corresponds to GC content and the inner light gray circle corresponds to the AT content. Different colors are used as a representation of distinctive genes within separate functional groups.

To further discern the genetic characteristic features of *S. guidongensis* and its close relatives within sect. *Glutinaria*, we employed comparative chloroplast genomics focusing on *S. guidongensis*, *S. nubicola*, *S. glutinosa*, and *S. chanryoenica*. Among these, the genomes of *S. guidongensis* and *S. nubicola* were sequenced in this study, while the plastomes of *S. glutinosa* and *S. chanryoenica* were sourced from GenBank. The complete plastomes of *S. guidongensis* and *S. nubicola* were assembled with no gaps (**Figure 5**). The full length of the two plastomes are 151,223 bp and 151,518 bp, respectively. Both plastomes exhibited the typical quadripartite structure, consisting of a pair of IRs (25,570–25,605 bp) separated by the LSC (82,434–82,903 bp) and SSC (17,628–17,649 bp) regions. The total GC content of the two genomes were both 38.0%. Both plastomes identically contain 88 protein-coding genes, 37 tRNAs, and eight rRNAs. Twenty genes have two copies, including 9 protein-coding genes (PCG) (*ndh*B, *rpl*2, *rpl*23, *rps*7, *rps*12, *rps*19, *ycf*1, *ycf*2, *ycf*15), 7 tRNA genes (*trn*A-UGC, *trn*I-CAU, *trn*I-GAU, *trn*L-CAA, *trn*N-GUU, *trn*R-ACG, *trn*V-GAC), and all 4 types of rRNA (rrn4.5, rrn5, rrn16, rrn23). Nine of the PCGs (*rps*16, *atp*F, *rpo*C1, *pet*B, *pet*D, *rpl*16, *rpl*2, *ndh*B and *ndh*A) contained one single intron, whereas three genes (*clp*P, *ycf*3, and *rps*12) contained two introns. The *ycf*1 gene in IRa was partially duplicated and formed a pseudogene.

#### 3.4.2 Variation at IR/SC boundaries hotspot regions identification

**Figure 6:**
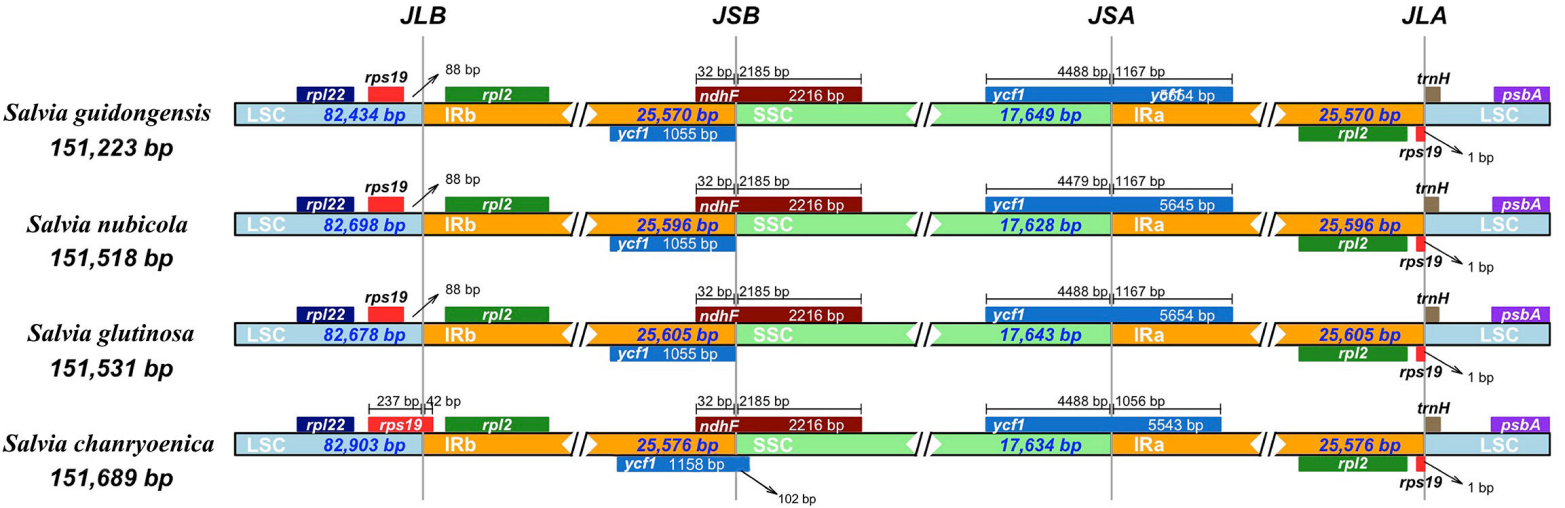
Comparative analysis of the junctions between the Large Single Copy (LSC), Inverted Repeats (IRs), and Small Single Copy (SSC) regions among four *Salvia* plastid genomes. The junctions include JLB (Junction of LSC and IRB), representing the junction between the LSC region and Inverted Repeat B (IRB); JSB (Junction of SSC and IRB), indicating the junction between the SSC region and IRB; JLA (Junction of LSC and IRA), denoting the junction between the LSC region and Inverted Repeat A (IRA); and JSA (Junction of SSC and IRA), signifying the junction between the SSC region and IRA.

**Figure 7:**
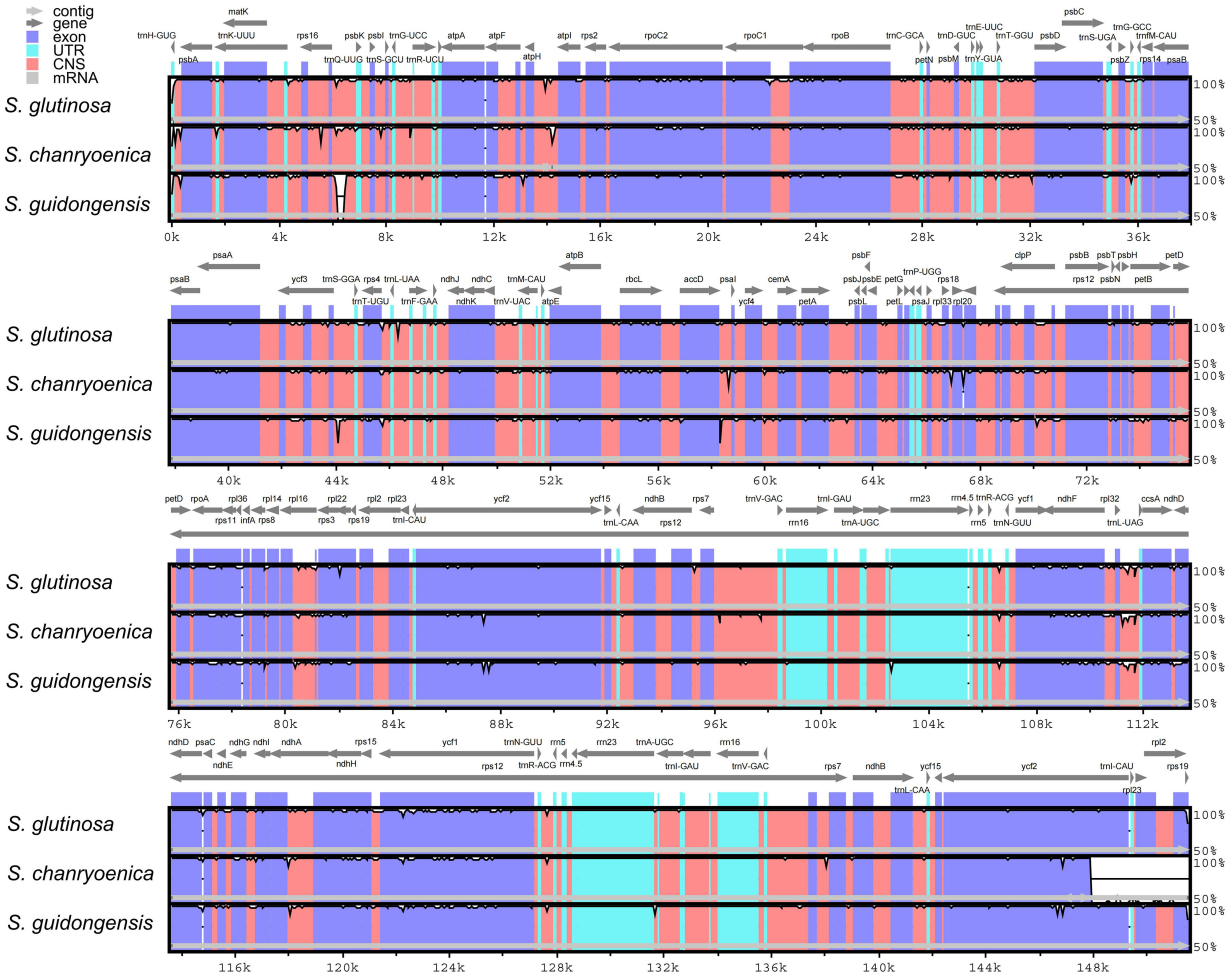
Comparative sequence identity analysis among four *Salvia* plastomes using *S. nubicola* as the reference. Regions highlighted include Conserved Non-coding Sequences (CNS) and Untranslated Regions (UTR).

Comparison of the four plastomes of *Salvia* revealed minor differences at the IR/LSC boundaries (**Figure 6**). At the IRa/LSC border, the space length from *rpl*19 to the border varied from 42 bp to 88 bp. At the IRb/LSC border, the space length from *psb*A to the border was all 82 bp. No variation was observed at the IR/LSC boundaries. All the IRb regions expanded 1,055 bp (with the exception of *S. chanryoenica*, which extended by 1,158 bp) into *ycf*1 and formed a pseudogene *Ψycf*1 in IRa by duplication. All IRa regions expanded into *ndh*F, causing a 32 bp overlap with *Ψycf*1. We analyzed the whole sequence divergence of the four *Salvia* plastomes using the mVISTA software with *S. glutinosa* as reference. After alignment, interspecific variations in sequences were discernible (**Figure 7**). Generally, the two IR regions exhibited lower sequence divergence compared to the LSC and SSC regions. Notably, within the 6,500 bp–6,870 bp range, *S. guidongensis* displayed a pronounced gap. Similarly, unique gaps and SNPs were evident in *S. guidongensis* between 44,000 bp to 48,000 bp and between 58,700 bp to 60,000 bp. Through interspecific comparisons, we observed that while the overall plastome structure and genes largely remained conserved, certain regions manifested significant divergence among species. These divergent regions offer valuable genomic insights for subsequent intrageneric evolutionary studies and species identification within the *Salvia* genus.

#### 3.4.3 Simple sequence repeats and codon usage

In terms of repeat type statistics (**Supplementary Table S5**), *S. chanryoenica* exhibited the highest total number of repeats at 115, followed by *S. nubicola* (108), *S. glutinosa* (106), and *S. guidongensis* (98). All four species demonstrated a consistent absence of reverse and complementary repeats. However, they varied in the number of forward repeats, with *S. chanryoenica* having the most (23) and *S. guidongensis* the least (19). When considering SSRs, *S. chanryoenica* again led with 45, while *S. guidongensis* had the fewest at 40. Tandem repeats were relatively consistent across the species, ranging from 18 in *S. guidongensis* to 24 in *S. chanryoenica*.

**Figure 8:**
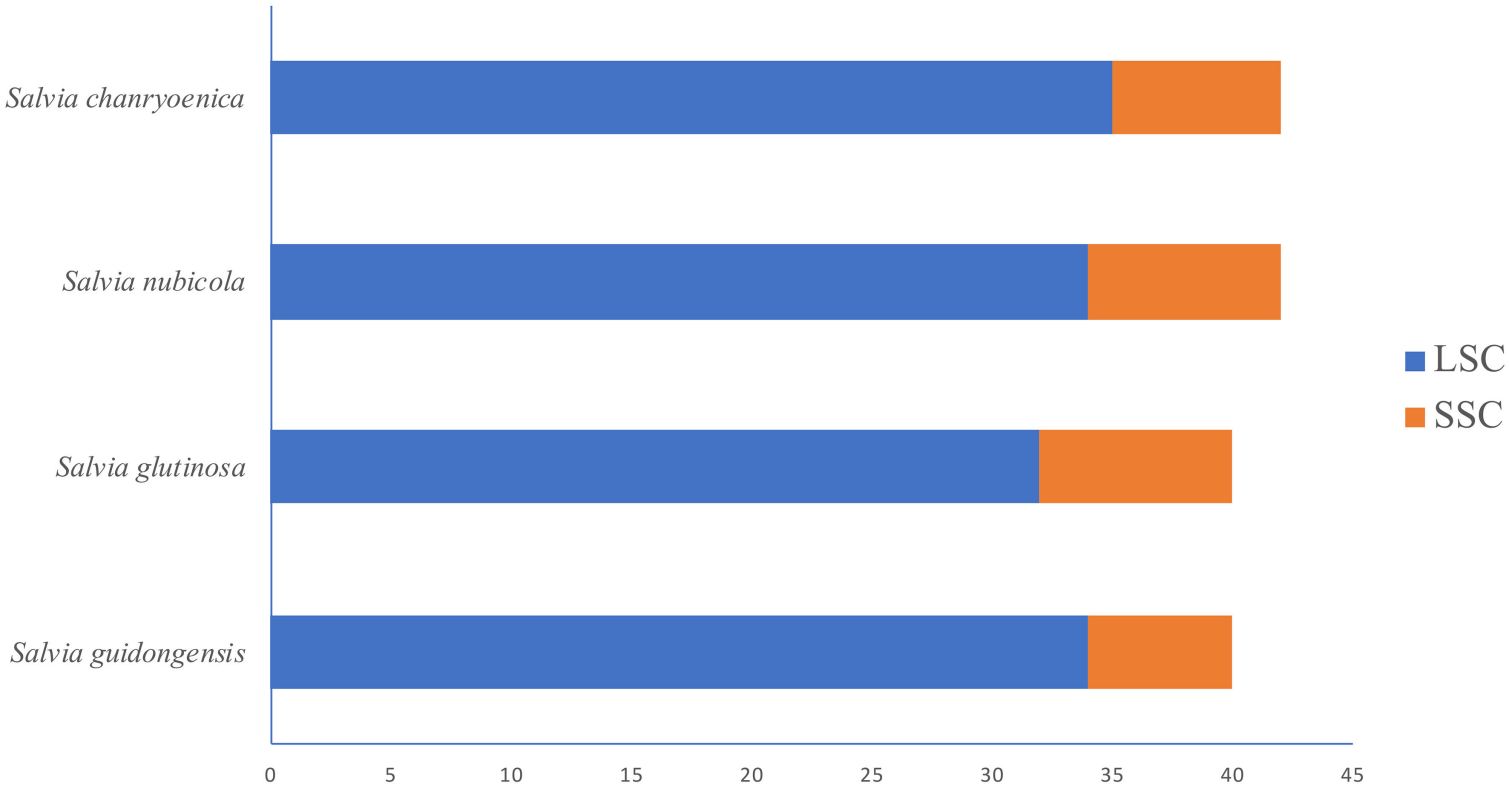
Horizontal bar chart illustrating the distribution of SSRs in the LSC and SSC regions of the plastomes for four *Salvia* species.

Regarding the distribution of SSRs in the plastomes (**Figure 8**; **Supplementary Table S6**), all four species showed SSRs exclusively in the LSC and SSC regions, with none detected in the IRa and IRb regions. *S. chanryoenica* had the highest number of SSRs in the LSC region (35), while both *S. guidongensis* and *S. nubicola* had 34 each, and *S. glutinosa* had 32. In the SSC region, *S. nubicola* had the highest count at 8, closely followed by *S. glutinosa* and *S. chanryoenica* with 7 each, and *S. guidongensis* had the fewest at 6.

Comprehensive codon usage analyses were conducted to discern potential evolutionary implications and species-specific adaptations. RSCU analysis (**Supplementary Table S7**) highlighted a distinct preference in *S. guidongensis* for the termination codon UAA, with an RSCU value of 1.8462. While certain codons, such as GCU for Alanine, were universally favored, the heightened affinity for UAA in *S. guidongensis* suggests unique evolutionary pressures. ΔRSCU values (**Supplementary Table S8**) further emphasized species-specific biases. *S. guidongensis*, *S. glutinosa*, and *S. nubicola* all showed a preference for GCA. In contrast, *S. chanryoenica* uniquely leaned towards GCA, CGA, and UGU. The pronounced ΔRSCU value for AUU in *S. guidongensis* underscores its genomic distinctiveness. Correlation analyses (**Supplementary Table S9**) across the species revealed a consistent, strong correlation between GC1 and GC_all, with coefficients ranging from 0.847 to 0.850. Additionally, a significant association between GC3 and ENC was observed in all species, hinting at a codon bias influenced by the GC content at the third codon position. The distribution of ENC ratios (**Supplementary Table S10**) further elucidated codon usage bias. All species, predominantly clustered around the 0.05~0.15 class range, suggesting potential shared evolutionary pressures or ancestral genomic traits. While the *Salvia* species exhibit overlapping genomic characteristics, the distinct codon preferences observed in *S. guidongensis* underscore its genomic differentiation. These unique attributes provide additional evidence supporting the establishment of *S. guidongensis* as a distinct species within sect. *Glutinaria*.

## 4 Discussion

The discovery of *S. guidongensis* and its subsequent morphological comparison, evolutionary phylogenetics, and plastid genomic characterization have enriched our comprehension of distribution and evolutionary trajectory within the East Asian *Salvia*, particularly in sect. *Glutinaria*.

Morphologically, *S. guidongensis* is characterized by ovate to elliptic leaves, significantly longer in both blade and petiole length than those of its congeners. This leaf morphology may represent an adaptation to the specific light conditions of its habitat. The larger leaf surface area is likely advantageous in the understory of subtropical broad-leaved forests, where light is a limiting factor (Rundel et al., 2020). This adaptation could be a response to evolutionary pressure to maximize photosynthesis in shaded environments (Malhado et al., 2009).

The dual floral colors, pale purple and pale yellow, suggest a unique pollination strategy. The variation in flower color within the same population could be an evolutionary response to attract a diverse range of pollinators, thereby increasing the chances of successful pollination in habitats with variable pollinator availability. These morphological intricacies often suggest evolutionary adaptations to specific ecological niches or pollinator diversification (Krishna and Keasar, 2018; Kriebel et al., 2020). Although flower color is generally seen as an adaptation to pollinator visual perception (Stanton, 1987; Niovi Jones and Reithel, 2001; Sobral et al., 2015), several studies have proposed that mechanisms beyond pollinator preferences might be at play in maintaining intraspecific floral color diversity. For example, neutral processes, or a lack of selection, could also perpetuate such variation (Sapir et al., 2021). The floral color dichotomy in this species warrants further exploration into the underlying mechanisms.

The multi-branched upright stem of *S. guidongensis*, in contrast to the unbranched stems of species such as *S. glabrescens* and *S. chanryoenica*, indicates a growth strategy adapted to its environment. The branching pattern could be an evolutionary response to effectively compete for light in its forest understory habitat (Kudo et al., 2008; Collet et al., 2011). This structural trait might also support the plant’s reproductive strategy by increasing flower numbers and thus potential for pollination (Lemmon et al., 2016).

From a plastid genomics perspective, *S. guidongensis* exhibits discernible differentiation from both western and eastern counterparts within the sect. *Glutinaria*, evident in SNPs, indels, and repetitive sequences (**Figures 6**–**8**). An intricate examination of codon preferences reveals a bias towards UAA and AUU, hinting at unique evolutionary pressures this species might have faced. Such genetic singularity accentuates the profound evolutionary divergence *S. guidongensis* has undergone relative to its congeners.

The geographical discovery of *S. guidongensis* is particularly significant as it fills a critical gap in the previously recognized disjunct distribution within sect. *Glutinaria.* This discovery establishes a crucial taxonomic connection, elucidating the phylogenetic and evolutionary relationships between the western and eastern taxa of the section. Specifically, while *S. glutinosa* and *S. nubicola* span regions from the Himalayas to Europe, the remaining five species are exclusively found in Japan, the Korean Peninsula, or Taiwan Island, China (Hu et al., 2018). Although recent identification, such as *S. nipponica* var. *zhejiangensis* in eastern mainland China (Wang et al., 2017), have expanded the known range of this section, they have not fully explained its distribution pattern. The presence of *S. guidongensis* in a region dominated by relict evergreen broad-leaved species (Tang et al., 2018) not only provides critical insights into the biogeographic connections between the European-Central Asian and Sino-Japanese floras but also highlights the adaptive responses of *Salvia* to the climatic and environmental shifts of the Late Miocene. The origin and divergence of *S. guidongensis* during this period, a time of significant environmental transformation in East Asia marked by open landscape expansion and intensified East Asian monsoon climate (Li et al., 2015), were likely influenced by the gradual cooling within an overall warm and humid climate, followed by more pronounced cooling episodes towards the end of the Miocene (Li et al., 2020). These climatic transitions were likely instrumental in the speciation of *S. guidongensis*.

Lastly, the conservation implications of *S. guidongensis* are paramount. Its confined distribution to a singular location in Guidong County and its limited population size underscore the urgency of conservation efforts. Amidst escalating environmental and anthropogenic threats, prioritizing its preservation becomes essential, not merely to preserve its genetic diversity but also to retain the evolutionary narratives it encapsulates.

## Data availability statement

The datasets presented in this study can be found in online repositories. The names of the repository/repositories and accession number(s) can be found in the article/**Supplementary Material**.

## Author contributions

YH: Conceptualization, Data curation, Funding acquisition, Investigation, Project administration, Resources, Visualization, Writing – original draft, Writing – review & editing. ZQ: Conceptualization, Data curation, Funding acquisition, Investigation, Project administration, Software, Visualization, Writing – original draft, Writing – review & editing. JF: Data curation, Formal analysis, Methodology, Validation, Visualization, Writing – review & editing. BG: Conceptualization, Funding acquisition, Investigation, Project administration, Resources, Writing – review & editing. CH: Investigation, Project administration, Resources, Supervision, Writing – review & editing. YF: Data curation, Formal analysis, Software, Validation, Writing – review & editing. JW: Writing – review & editing. PW: Data curation, Resources, Validation, Writing – review & editing. TI: Formal analysis, Methodology, Resources, Writing – review & editing. GK: Formal analysis, Methodology, Resources, Writing – review & editing. PL: Investigation, Project administration, Resources, Supervision, Writing – review & editing. YW: Conceptualization, Funding acquisition, Investigation, Project administration, Resources, Writing – review & editing.
