# Supplementary material for: Salvia guidongensis sp. nov.: unraveling a critical evolutionary link in East Asian Salvia from Central China integrating morphology, phylogeny, and plastid genomics
Source: Front Plant Sci. 2024 Mar 5;15:1332443. doi: 10.3389/fpls.2024.1332443 (PMC10948445; doi:10.3389/fpls.2024.1332443)
Supplement: Supplementary file 1 [file DataSheet_1.docx]

Supplementary Material

*Salvia guidongensis* sp. nov.: Unraveling a Critical Evolutionary Link in East Asian *Salvia* from Central China Integrating Morphology, Phylogeny, and Plastid Genomics

Yan-Bo Huang^1^, Zhe-Chen Qi^2*^, Jie-Ying Feng^1,2^, Bin-Jie Ge^1*^, Cun-Zhong Huang^3^, Yu-Qing Feng^2^, Jing Wu^4^, Pu-Rui Wei^5^, Takuro Ito^6^, Goro Kokubugata^7^, Pan Li^4*^, Yu-Kun Wei^1,8*^

*** Correspondence:** Zhe-Chen Qi: [zqi@zstu.edu.cn](mailto:zqi@zstu.edu.cn); Bin-Jie Ge: [gebinjie@csnbgsh.cn](mailto:gebinjie@csnbgsh.cn); Pan Li: [panli@zju.edu.cn](mailto:panli@zju.edu.cn); Yu-Kun Wei: [wyk@shbg.org](mailto:wyk@shbg.org;).

**Contents**

[1 Supplementary Figures 2](#_Toc26386)

[2 Supplementary Tables 4](#_Toc8828)

# Supplementary Figures


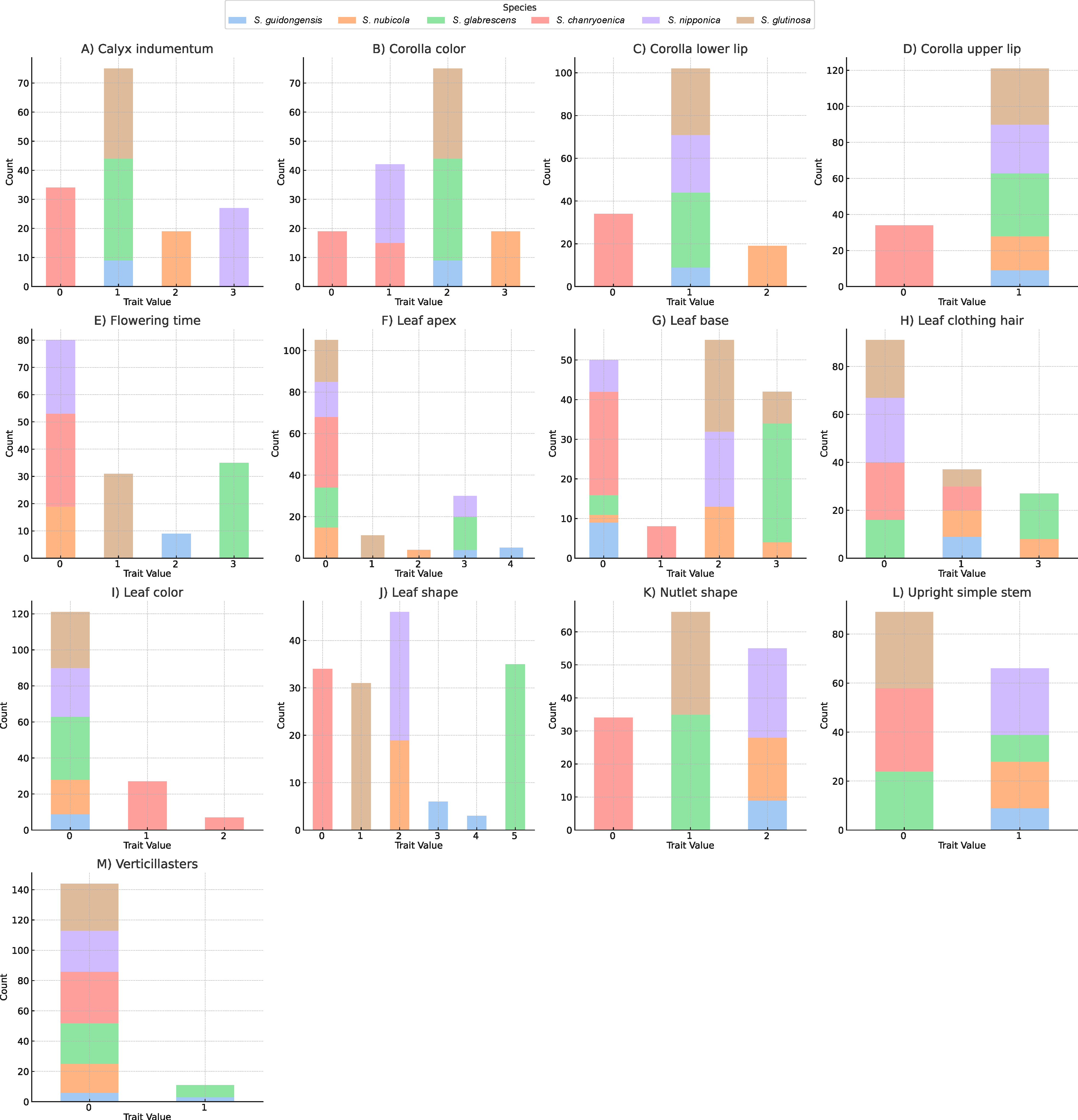


**Figure S1**. Panel chart depicting counts of character states for each analyzed qualitative trait across six *Salvia* species in sect. *Glutinaria*. The chart illustrates the distribution of trait values for each species, represented by different colors. For specific information regarding the values of each trait, refer to Table S4.


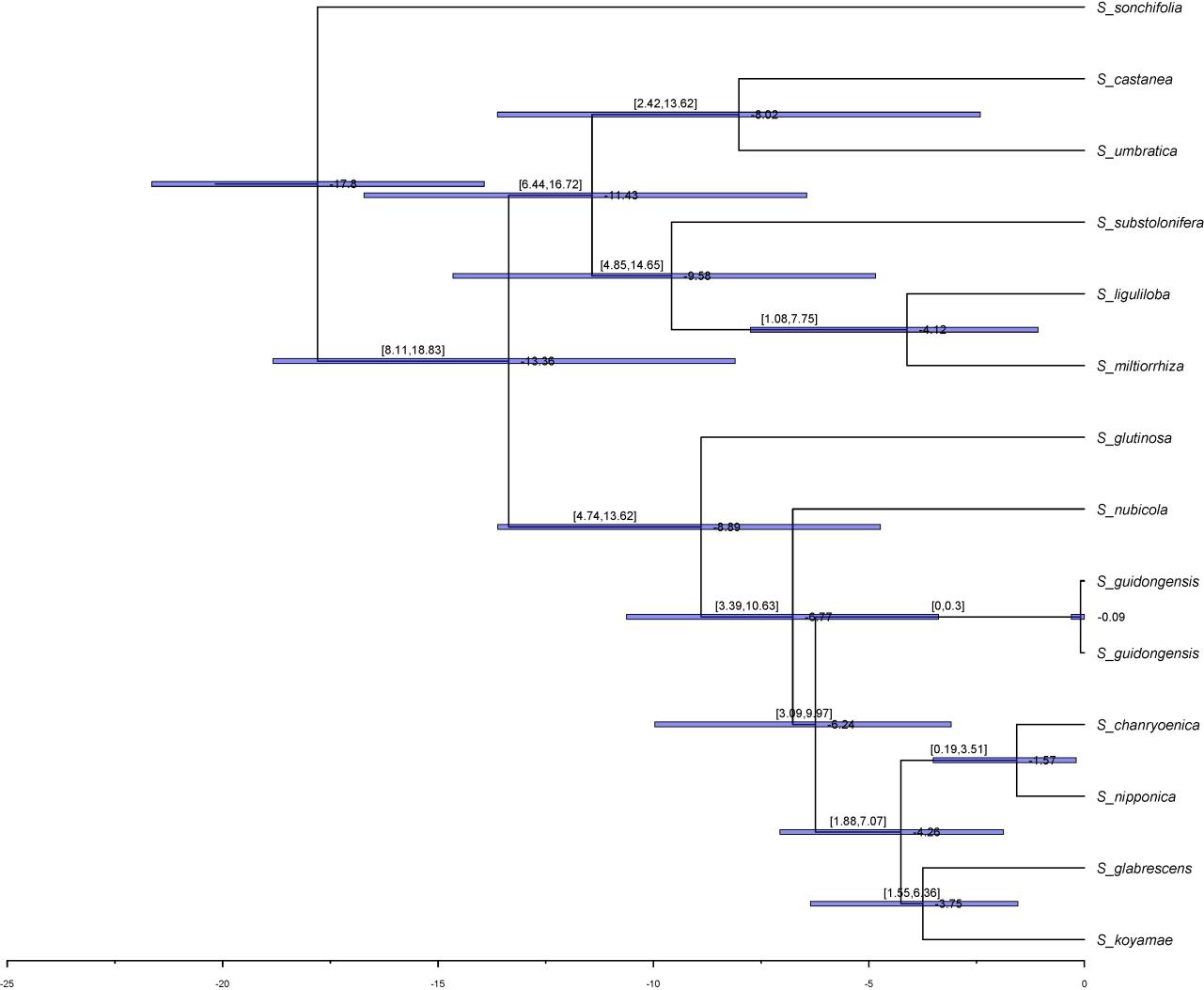


**Figure S2**. BEAST chronogram of *S. guidongensis* alongside 12 other *Salvia* species, illustrating a clade within subg. *Glutinaria* clade. Median divergence time and 95% HPDs (blue bars) are indicated on each node and branch, respectively.

# Supplementary Tables

**Table S1**. Taxa included in the present study with voucher information and GenBank accession numbers for the sequences of *mat*K, *ndh*A, *ndh*F, *psb*A-*trn*H, *rbc*L, *rpl*16, *trn*L-F, and *ycf*1-*rps*15. Missing sequences are indicated by a dash (-). Accession numbers in bold are newly generated sequences in this study.

| **Species** | **Voucher** | **Location** | ***matK*** | ***rbcL*** | ***ndhA*** | ***ndhF*** | ***rpl16*** | ***trnL-F*** | ***psbA-trnH*** | ***ycf1-rps15*** | **Plastome** |
| --- | --- | --- | --- | --- | --- | --- | --- | --- | --- | --- | --- |
| *Salvia guidongensis* C.Z. Huang, Yan.B. Huang, B.J. Ge, & Z.C. Qi, sp. nov. | S1799 (CSH) | Guidong County, Hunan, China | **OR732592** | **OR771031** | **OR771053** | **OR771064** | **OR771042** | **OR771075** | **OR771092** | **OR771107** | **OR767373** |
| *Salvia guidongensis* C.Z. Huang, Yan.B. Huang, B.J. Ge, & Z.C. Qi, sp. nov. | S1800 (CSH) | Guidong County, Hunan, China | **OR732593** | **OR771032** | **OR771054** | **OR771065** | **OR771043** | **OR771076** | **OR771093** | **OR771108** |  |
| *Salvia sonchifolia* C.Y.Wu | S0683 (CSH) | Xichou, Yunnan, China | **OR732598** | **OR771037** | **OR771059** | **OR771070** | **OR771048** | **OR771081** | **OR771098** | **OR771100** |  |
| *Salvia castanea* Diels | S0638 (CSH) | Lijiang, Yunan, China | **OR732590** | **OR771029** | **OR771051** | **OR771062** | **OR771040** | **OR771073** | **OR771096** | **OR771102** |  |
| *Salvia umbratica* Hance | S0421 (CSH) | Beijing, China | **OR732600** | **OR771039** | **OR771061** | **OR771072** | **OR771050** | **OR771083** | **OR771097** | **OR771103** |  |
| *Salvia substolonifera* E.Peter | S0477 (CSH) | Leibo, Sichuan, China | **OR732599** | **OR771038** | **OR771060** | **OR771071** | **OR771049** | **OR771082** | **OR771099** | **OR771104** |  |
| *Salvia liguliloba* Sun | S0503 (CSH) | Linan, Zhejiang, China | **OR732594** | **OR771033** | **OR771055** | **OR771066** | **OR771044** | **OR771077** | **OR771094** | **OR771101** |  |
| *Salvia miltiorrhiza* Bunge | S0424 (CSH) | Beijing, China | **OR732595** | **OR771034** | **OR771056** | **OR771067** | **OR771045** | **OR771078** | **OR771095** | **OR771110** |  |
| *Salvia glutinosa* L. | S1031 (CSH) | Cult. at Royal Botanical Garden, UK | **OR732591** | **OR771030** | **OR771052** | **OR771063** | **OR771041** | **OR771074** | **OR771091** | **OR771105** | NC067736 |
| *Salvia nubicola* Wall. ex Sweet | S0986 (CSH) | Xizang, China | **OR732597** | **OR771036** | **OR771058** | **OR771069** | **OR771047** | **OR771080** | **OR771090** | **OR771106** | **OR772298** |
| *Salvia chanryoenica* Nakai | MH261357 (KH) | Mt. Sobaek, South Korea | AYO45821 | AYO45847 | AYO45893 | AYO45885 | AYO45875 | NC_040121 | NC_040121 | NC_040121 | MH261357 |
| *Salvia nipponica* Miq. | S1005 (CSH) | Kanto Area, Honshu, Japan | **OR732596** | **OR771035** | **OR771057** | **OR771068** | **OR771046** | **OR771079** | **OR771089** | **OR771109** |  |
| *Salvia glabrescens* (Franch. & Sav.) Makino | 2019-26 | NA | NC_067735.1 | NC_067735.1 | NC_067735.1 | NC_067735.1 | NC_067735.1 | NC_067735.1 | NC_067735.1 | NC_067735.1 |  |
| *Salvia koyamae* Makino | ATakano090704-2 (HYO) | cult. at Kyoto, Kameoka, Kameyama | - | AB541128 | - | - | - | AB541142 | MG823960 | LC060540 |  |

**Table S2.** PCR Primers used in this study.

| **Regions** | **Primer sequence 5’–3’** | **References** |
| --- | --- | --- |
| *rbc*L | ATGTCACCACAAACAGAAACTAAAGCAAGT | Hu et al. (2018) |
|  | CTTCACAAGCAGCAGCTAGTTCAGGACTCC |  |
| *mat*K | CTATATCCACTTATCTTTCAGGAGT | Bendiksby et al. (2011) |
|  | CAAGAAAGTCGAAGTATATACTTTA |  |
| *ndh*F | TCYGCTTCTGGTAAATCAAA | Scarcelli et al. (2011) |
|  | AATATCTCTACGTGYGATTCG |  |
| *ndh*A | TCYGCTTCTGGTAAATCAAA | Shaw et al.(2007) |
|  | AATATCTCTACGTGYGATTCG |  |
| *rpl16* | CCYYTCATTCTTCCTCTATGTTG | Scarcelli et al. (2011) |
|  | TGCTTAGTGTGTGACTCGTT |  |
| *psb*A-*trn*H | GTTATGCATGAACGTAATGCTC | Hu et al. (2018) |
|  | CGCGCATGGTGGATTCACAAATC |  |
| *trn*L-F | CGAAATCGGTAGACGCTACG | Hu et al. (2018) |
|  | ATTTGAACTGGTGACACGAG |  |
| *ycf*1-*rps*15 | CTTGTATGRATCGTTATTGKTTTG | Hu et al. (2018) |
|  | CAATTYCAAATGTGAAGTAAGTCTCC |  |

**Table S3**. Comparison of qualitative and quantitative traits between *S. guidongensis* and its close relatives in sect. *Glutinaria*. The information for 'populations/total individuals (min-max per population)' is provided alongside each species name.

| Characters | *S. guidongensis* 1/34 | *S. nubicola* 2/31(15–16) | *S. glabrescens* 1/19 | *S. chanryoenica* 1/9 | *S.* *nipponica* 3/27(7–10) | *S. glutinosa* 2/35(12–13) |
| --- | --- | --- | --- | --- | --- | --- |
| Plant height | 50.0–88.0 cm  (Mean: 76.0, SD: 7.7) | 100.0–125.0 cm  (Mean: 110.0, SD: 8.6) | 23.7–48.0 cm  (Mean: 33.0, SD: 6.8) | 38.0–82.0 cm  (Mean: 52.8, SD: 17.0) | 20.0–40.0 cm  (Mean: 35.0, SD: 6.5) | 40.0–120.0 cm  (Mean: 75.9, SD: 27.7) |
| Upright simple stem | multi-branched | multi-branched | Unbranched | Unbranched | Unbranched | Unbranched or multi-branched |
| Petiole length | 9.0–14.0 cm  (Mean: 12.1, SD: 1.0) | 5.0–13.0 cm  (Mean: 7.0, SD: 1.7) | 3.4–8.6 cm  (Mean: 6.0, SD: 1.6) | 3.2–12.4 cm  (Mean: 6.8, SD: 2.5) | 4.1–11.1 cm  (Mean: 8.7, SD: 2.0) | 3.9–8.6 cm  (Mean: 5.9, SD: 1.2) |
| Leaf blade | Length: 16.2–25.0 cm (Mean: 21.5, SD: 2.4)  Width: 7.8–13.0 cm (Mean: 11.0, SD: 1.6) | Length: 10.4–18.4 cm (Mean: 12.8, SD: 1.9)  Width: 5.4–10.0 cm (Mean: 7.6, SD: 1.1) | Length: 6.3–12.7 cm (Mean: 8.6, SD: 1.7)  Width: 4.2–5.5 cm (Mean: 5.1, SD: 0.4) | Length: 8.4–12.6 cm (Mean: 10.0, SD: 1.2)  Width: 5.9–9.2 cm (Mean: 7.8, SD: 1.1) | Length: 5.0–12.4 cm (Mean: 8.5, SD: 1.9)  Width: 4.4–11.2 cm (Mean: 7.6, SD: 1.8) | Length: 7.9–17.6 cm (Mean: 10.3, SD: 2.9)  Width: 4.0–9.9 cm (Mean: 6.3, SD: 1.7) |
| Leaf shape | Long elliptic | Triangular-ovate | Hastate | Oval, elliptic | Hastate | Oblong |
| Leaf apex | Acuminate | Acuminate to acute | Acuminate to caudate | Subacute to cuspidate | Acuminate to cuspidate | Acuminate to cuspidate |
| Leaf base | Cordate, rounded | Hastate, sagittate | Hastate, sagittate or cordate | Cordate | Hastate or cordate | Hastate or cordate |
| Leaf color | Dark green or yellow green | Green | Green | Green | Green | Green |
| Leaf [clothing hair](javascript:;) | Sparsely villous or glabrous | Sparsely villous or glabrous | Glabrous, leaf veins sparsely hairy | Glabrous | Sparsely villous | Glabrous, leaf veins sparsely hairy |
| Verticillasters | 6 flowered | 6 flowered | 6 flowered | 2–6 flowered | 6 flowered | 4–6 flowered |
| Pedicel length | 2.0–3.8 mm  (Mean: 2.9, SD: 0.6) | 3.4–5.2 mm  (Mean: 4.0, SD: 0.5) | 2.5–6.0 mm  (Mean: 3.8, SD: 1.1) | 5.3–6.0 mm  (Mean: 5.6, SD: 0.2) | 3.0–9.0 mm  (Mean: 4.4, SD: 1.7) | 2.9–5.1 mm  (Mean: 3.9, SD: 0.8) |
| Calyx length | 10.4–13.5 mm  (Mean: 11.4, SD: 0.8) | 8.9–11.7 mm  (Mean: 10.1, SD: 0.7) | 6.0–11.0 mm  (Mean: 8.7, SD: 1.3) | 8.0–14.0 mm  (Mean: 10.6, SD: 1.7) | 7.0–17.0 mm  (Mean: 12.3, SD: 2.5) | 9.6–13.0 mm  (Mean: 11.5, SD: 1.1) |
| Calyx indumentum | Densely glandular hairy | Pubescent, long glandular hairy | Obviously and long strigose on edges | Pubescent, long glandular hairy | Sparsely hirsute | Pubescent, long glandular hairy |
| Corolla color | Pale purple, pale yellow | Yellow | Violet | Yellow | Pale yellow | Yellow |
| Corolla length | 23.1–27.9 mm  (Mean: 25.4, SD: 1.1) | 19.5–25.8 mm  (Mean: 23.7, SD: 1.8) | 20.0–28.2 mm  (Mean: 25.0, SD: 2.0) | 25.2–33.5 mm  (Mean: 30.0, SD: 2.4) | 25.0–35.0 mm  (Mean: 32.0, SD: 3.0) | 29.1–34.7 mm  (Mean: 32.5, SD: 1.8) |
| Corolla height | 15.9–20.4 mm  (Mean: 18.3, SD: 1.2) | 13.1–19.0 mm  (Mean: 16.5, SD: 1.5) | 5.5–20.0 mm  (Mean: 12.4, SD: 3.8) | 11.0–20.0 mm  (Mean: 18.4, SD: 2.8) | 10.1–18.0 mm  (Mean: 14.6, SD: 1.9) | 16.1–20.6 mm  (Mean: 18.7, SD: 1.2) |
| Corolla width | 7.6–9.1 mm  (Mean: 8.3, SD: 0.5) | 6.4–10.7 mm  (Mean: 8.9, SD: 1.0) | NA | NA | NA | 7.5–9.5 mm  (Mean: 8.5, SD: 0.7) |
| Corolla upper lip | Falcate, sparsely glandular hairy | Falcate, glandular pubescent | Falcate, glandular pubescent | Falcate, glandular pubescent | Falcate, glandular pubescent | Falcate, glandular pubescent |
| Corolla lower lip | Middle lobe sharply inward curl | Middle lobe curl outward | Middle lobe slightly curl inward | Middle lobe slightly curl inward | Middle lobe curl outward | Middle lobe curl outward |
| Stamen connective length | 10.5–12.5 mm  (Mean: 11.7, SD: 0.5) | 7.4–10.8 mm  (Mean: 9.7, SD: 0.8) | NA | NA | NA | 10.5–12.0 mm  (Mean: 11.3, SD: 0.5) |
| Filament length | 3.3–5.0 mm  (Mean: 4.3, SD: 0.5) | 3.4–5.0 mm  (Mean: 4.4, SD: 0.4) | NA | NA | NA | 4.2–5.3 mm  (Mean: 4.8, SD: 0.3) |
| Pistil length | 30.1–38.3 mm  (Mean: 33.2, SD: 1.5) | 24.1–29.0 mm  (Mean: 26.9, SD: 1.2) | NA | NA | NA | 33.3–38.0 mm  (Mean: 35.2, SD: 1.5) |
| Nutlet shape | Oblong, approximately globular | Elliptical | Obovate | Obovate | Obovate | Elliptical |
| Nutlet size | 2.8–3.1 mm  (Mean: 3.0, SD: 0.1) | 2.3–3.2 mm  (Mean: 2.9, SD: 0.2) | NA | NA | NA | 2.8–3.4 mm  (Mean: 3.1, SD: 0.2) |
| Flowering time | August to October | June to August | August to October | July to August | August to October | July to October |

**Table S4**. Encoding of qualitative morphological and ecological (Flowering time) characteristics for species in sect. *Glutinaria*

| Qualitative characters | States |
| --- | --- |
| Upright simple stem | 0-Multi-branched; 1-Unbranched |
| Leaf shape | 0-Long elliptic; 1-Triangular-ovate; 2-Hastate; 3-Oval; 4-Elliptic; 5-Oblong |
| Leaf apex | 0-Acuminate; 1-Acute; 2-Caudate; 3-Cuspidate; 4-Subacute |
| Leaf base | 0-Cordate; 1-Rounded; 2-Hastate; 3-Sagittate |
| Leaf color | 0-Green; 1-Dark green; 2-Yellow green |
| Leaf [clothing hair](javascript:;) | 0-Sparsely villous; 1-Glabrous; 2-Sparsely pilose; 3-Sparsely hairy on leaf veins |
| Verticillasters | 0-Flower six; 1-Flower ≤ six |
| Calyx indumentum | 0-Densely glandular hairy; 1-Pubescent, long glandular hairy; 2-Obviously and long strigose on edges; 3-Sparsely hirsute |
| Corolla color | 0-Pale purple; 1-Pale yellow; 2-Yellow; 3-Violet |
| Corolla upper lip | 0-Falcate, sparsely glandular hairy; 1-Falcate, glandular pubescent |
| Corolla lower lip | 0-Middle lobe sharply inward curl; 1-Middle lobe curl outward; 2-Middle lobe slightly curl inward |
| Nutlet shape | 0-Oblong, approximately globular; 1-Elliptical; 2-Obovate |
| Flowering time | 0-August to October; 1-June to August; 2-July to August; 3-July to October |

**Table S5.** Repeats type statistics of four *Salvia* species.

|  | Dispersed | | | | Palindro-mic repeats | SSR | Tandem Repeats | Total |
| --- | --- | --- | --- | --- | --- | --- | --- | --- |
| Species | **Forward repeats** | **Reverse repeats** | **Complim-entary repeats** | **Total** |  |  |  |  |
| *Salvia guidongensis* | 19 | 0 | 0 | 19 | 21 | 40 | 18 | 117 |
| *Salvia nubicola* | 21 | 0 | 0 | 21 | 23 | 43 | 21 | 129 |
| *Salvia glutinosa* | 21 | 0 | 0 | 21 | 24 | 40 | 21 | 127 |
| *Salvia chanryoenica* | 23 | 0 | 0 | 23 | 23 | 45 | 24 | 138 |

**Table S6**. Classification of simple sequence repeats (SSRs) in the chloroplast genomes of four *Salvia* species based on the distribution of SSRs.

| **Species** | **LSC** | **SSC** | **IRa** | **IRb** |
| --- | --- | --- | --- | --- |
| *Salvia guidongensis* | 34 | 6 | 0 | 0 |
| *Salvia glutinosa* | 32 | 8 | 0 | 0 |
| *Salvia nubicola* | 34 | 8 | 0 | 0 |
| *Salvia chanryoenica* | 35 | 7 | 0 | 0 |

**Table S7**. RSCU analysis of amino acids in chloroplast genome of 4 *Salvia* species.

| *Salvia guidongensis* | | | | |
| --- | --- | --- | --- | --- |
| AminoAcid | Symbol | Codon | Number | RSCU |
| * | Ter | UAG | 9 | 0.5193 |
| * | Ter | UGA | 11 | 0.6345 |
| * | Ter | UAA | 32 | 1.8462 |
| A | Ala | GCC | 186 | 0.6432 |
| A | Ala | GCA | 325 | 1.1236 |
| A | Ala | GCG | 135 | 0.4668 |
| A | Ala | GCU | 511 | 1.7668 |
| C | Cys | UGC | 55 | 0.4804 |
| C | Cys | UGU | 174 | 1.5196 |
| D | Asp | GAC | 171 | 0.4038 |
| D | Asp | GAU | 676 | 1.5962 |
| E | Glu | GAG | 267 | 0.4772 |
| E | Glu | GAA | 852 | 1.5228 |
| F | Phe | UUU | 804 | 1.3814 |
| F | Phe | UUC | 360 | 0.6186 |
| G | Gly | GGC | 162 | 0.452 |
| G | Gly | GGA | 569 | 1.5872 |
| G | Gly | GGG | 263 | 0.7336 |
| G | Gly | GGU | 440 | 1.2272 |
| H | His | CAU | 388 | 1.5336 |
| H | His | CAC | 118 | 0.4664 |
| I | Ile | AUA | 529 | 0.8967 |
| I | Ile | AUU | 885 | 1.5 |
| I | Ile | AUC | 356 | 0.6033 |
| K | Lys | AAA | 853 | 1.5164 |
| K | Lys | AAG | 272 | 0.4836 |
| L | Leu | CUA | 293 | 0.8058 |
| L | Leu | CUG | 147 | 0.4044 |
| L | Leu | CUU | 466 | 1.2816 |
| L | Leu | UUG | 448 | 1.2318 |
| L | Leu | CUC | 127 | 0.3492 |
| L | Leu | UUA | 701 | 1.9278 |
| M | Met | AUG | 480 | 1 |
| N | Asn | AAU | 782 | 1.5608 |
| N | Asn | AAC | 220 | 0.4392 |
| P | Pro | CCC | 181 | 0.8192 |
| P | Pro | CCA | 247 | 1.1176 |
| P | Pro | CCG | 130 | 0.5884 |
| P | Pro | CCU | 326 | 1.4752 |
| Q | Gln | CAA | 591 | 1.5472 |
| Q | Gln | CAG | 173 | 0.4528 |
| R | Arg | CGA | 282 | 1.3524 |
| R | Arg | CGG | 99 | 0.4746 |
| R | Arg | CGU | 272 | 1.3044 |
| R | Arg | AGG | 122 | 0.585 |
| R | Arg | AGA | 374 | 1.794 |
| R | Arg | CGC | 102 | 0.489 |
| S | Ser | UCC | 255 | 0.9672 |
| S | Ser | AGU | 329 | 1.248 |
| S | Ser | UCG | 148 | 0.5616 |
| S | Ser | AGC | 87 | 0.33 |
| S | Ser | UCA | 308 | 1.1682 |
| S | Ser | UCU | 455 | 1.7256 |
| T | Thr | ACG | 104 | 0.4036 |
| T | Thr | ACA | 311 | 1.2064 |
| T | Thr | ACU | 418 | 1.6216 |
| T | Thr | ACC | 198 | 0.768 |
| V | Val | GUC | 132 | 0.4556 |
| V | Val | GUA | 444 | 1.5324 |
| V | Val | GUG | 154 | 0.5316 |
| V | Val | GUU | 429 | 1.4804 |
| W | Trp | UGG | 379 | 1 |
| Y | Tyr | UAU | 618 | 1.637 |
| Y | Tyr | UAC | 137 | 0.363 |
| *Salvia glutinosa* | | | | |
| AminoAcid | Symbol | Codon | Number | RSCU |
| * | Ter | UAA | 31 | 1.7886 |
| * | Ter | UAG | 10 | 0.5769 |
| * | Ter | UGA | 11 | 0.6345 |
| A | Ala | GCG | 136 | 0.4704 |
| A | Ala | GCA | 324 | 1.1212 |
| A | Ala | GCC | 188 | 0.6504 |
| A | Ala | GCU | 508 | 1.7576 |
| C | Cys | UGC | 53 | 0.465 |
| C | Cys | UGU | 175 | 1.535 |
| D | Asp | GAC | 171 | 0.4038 |
| D | Asp | GAU | 676 | 1.5962 |
| E | Glu | GAA | 853 | 1.526 |
| E | Glu | GAG | 265 | 0.474 |
| F | Phe | UUC | 360 | 0.619 |
| F | Phe | UUU | 803 | 1.381 |
| G | Gly | GGC | 162 | 0.4508 |
| G | Gly | GGU | 442 | 1.2296 |
| G | Gly | GGA | 570 | 1.5856 |
| G | Gly | GGG | 264 | 0.7344 |
| H | His | CAU | 385 | 1.5278 |
| H | His | CAC | 119 | 0.4722 |
| I | Ile | AUC | 356 | 0.6048 |
| I | Ile | AUU | 885 | 1.5033 |
| I | Ile | AUA | 525 | 0.8919 |
| K | Lys | AAG | 271 | 0.4848 |
| K | Lys | AAA | 847 | 1.5152 |
| L | Leu | UUA | 701 | 1.926 |
| L | Leu | CUA | 293 | 0.8052 |
| L | Leu | CUG | 146 | 0.4008 |
| L | Leu | UUG | 448 | 1.2306 |
| L | Leu | CUU | 467 | 1.2828 |
| L | Leu | CUC | 129 | 0.3546 |
| M | Met | AUG | 479 | 1 |
| N | Asn | AAU | 780 | 1.5584 |
| N | Asn | AAC | 221 | 0.4416 |
| P | Pro | CCG | 125 | 0.5656 |
| P | Pro | CCA | 250 | 1.1312 |
| P | Pro | CCC | 183 | 0.828 |
| P | Pro | CCU | 326 | 1.4752 |
| Q | Gln | CAA | 591 | 1.541 |
| Q | Gln | CAG | 176 | 0.459 |
| R | Arg | CGG | 98 | 0.4698 |
| R | Arg | CGC | 104 | 0.4986 |
| R | Arg | AGA | 376 | 1.8018 |
| R | Arg | CGU | 274 | 1.3128 |
| R | Arg | CGA | 280 | 1.3416 |
| R | Arg | AGG | 120 | 0.5748 |
| S | Ser | UCC | 258 | 0.9798 |
| S | Ser | UCU | 452 | 1.7166 |
| S | Ser | UCG | 149 | 0.5658 |
| S | Ser | AGC | 88 | 0.3342 |
| S | Ser | AGU | 327 | 1.242 |
| S | Ser | UCA | 306 | 1.1622 |
| T | Thr | ACA | 309 | 1.2 |
| T | Thr | ACG | 103 | 0.4 |
| T | Thr | ACC | 199 | 0.7728 |
| T | Thr | ACU | 419 | 1.6272 |
| V | Val | GUG | 154 | 0.5324 |
| V | Val | GUA | 445 | 1.5384 |
| V | Val | GUU | 427 | 1.4764 |
| V | Val | GUC | 131 | 0.4528 |
| W | Trp | UGG | 378 | 1 |
| Y | Tyr | UAU | 615 | 1.6378 |
| Y | Tyr | UAC | 136 | 0.3622 |
| *Salvia nubicola* | | | | |
| AminoAcid | Symbol | Codon | Number | RSCU |
| * | Ter | UAA | 32 | 1.8462 |
| * | Ter | UGA | 11 | 0.6345 |
| * | Ter | UAG | 9 | 0.5193 |
| A | Ala | GCC | 189 | 0.6552 |
| A | Ala | GCU | 502 | 1.74 |
| A | Ala | GCA | 325 | 1.1264 |
| A | Ala | GCG | 138 | 0.4784 |
| C | Cys | UGU | 175 | 1.5284 |
| C | Cys | UGC | 54 | 0.4716 |
| D | Asp | GAU | 677 | 1.6004 |
| D | Asp | GAC | 169 | 0.3996 |
| E | Glu | GAG | 266 | 0.475 |
| E | Glu | GAA | 854 | 1.525 |
| F | Phe | UUC | 363 | 0.6232 |
| F | Phe | UUU | 802 | 1.3768 |
| G | Gly | GGC | 162 | 0.4504 |
| G | Gly | GGU | 444 | 1.234 |
| G | Gly | GGA | 568 | 1.5788 |
| G | Gly | GGG | 265 | 0.7368 |
| H | His | CAC | 117 | 0.4624 |
| H | His | CAU | 389 | 1.5376 |
| I | Ile | AUC | 358 | 0.6072 |
| I | Ile | AUU | 882 | 1.4958 |
| I | Ile | AUA | 529 | 0.897 |
| K | Lys | AAG | 270 | 0.4812 |
| K | Lys | AAA | 852 | 1.5188 |
| L | Leu | CUA | 292 | 0.8016 |
| L | Leu | UUG | 450 | 1.2354 |
| L | Leu | CUC | 128 | 0.3516 |
| L | Leu | CUU | 468 | 1.2852 |
| L | Leu | UUA | 700 | 1.9224 |
| L | Leu | CUG | 147 | 0.4038 |
| M | Met | AUG | 481 | 1 |
| N | Asn | AAU | 785 | 1.5606 |
| N | Asn | AAC | 221 | 0.4394 |
| P | Pro | CCA | 249 | 1.1256 |
| P | Pro | CCC | 181 | 0.818 |
| P | Pro | CCU | 328 | 1.4824 |
| P | Pro | CCG | 127 | 0.574 |
| Q | Gln | CAA | 593 | 1.5504 |
| Q | Gln | CAG | 172 | 0.4496 |
| R | Arg | AGA | 373 | 1.7934 |
| R | Arg | CGG | 99 | 0.4758 |
| R | Arg | AGG | 118 | 0.5676 |
| R | Arg | CGU | 272 | 1.3074 |
| R | Arg | CGC | 104 | 0.4998 |
| R | Arg | CGA | 282 | 1.356 |
| S | Ser | UCA | 307 | 1.1622 |
| S | Ser | UCC | 254 | 0.9618 |
| S | Ser | AGU | 331 | 1.2528 |
| S | Ser | UCG | 149 | 0.564 |
| S | Ser | UCU | 457 | 1.7298 |
| S | Ser | AGC | 87 | 0.3294 |
| T | Thr | ACU | 418 | 1.6216 |
| T | Thr | ACC | 199 | 0.772 |
| T | Thr | ACA | 311 | 1.2064 |
| T | Thr | ACG | 103 | 0.3996 |
| V | Val | GUG | 154 | 0.532 |
| V | Val | GUC | 131 | 0.4524 |
| V | Val | GUU | 429 | 1.482 |
| V | Val | GUA | 444 | 1.5336 |
| W | Trp | UGG | 379 | 1 |
| Y | Tyr | UAC | 136 | 0.3608 |
| Y | Tyr | UAU | 618 | 1.6392 |
| *Salvia chanryoenica* | | | | |
| AminoAcid | Symbol | Codon | Number | RSCU |
| * | Ter | UGA | 11 | 0.6471 |
| * | Ter | UAA | 31 | 1.8234 |
| * | Ter | UAG | 9 | 0.5295 |
| A | Ala | GCA | 317 | 1.1272 |
| A | Ala | GCG | 133 | 0.4728 |
| A | Ala | GCU | 489 | 1.7388 |
| A | Ala | GCC | 186 | 0.6612 |
| C | Cys | UGC | 52 | 0.4882 |
| C | Cys | UGU | 161 | 1.5118 |
| D | Asp | GAU | 651 | 1.5898 |
| D | Asp | GAC | 168 | 0.4102 |
| E | Glu | GAG | 252 | 0.4714 |
| E | Glu | GAA | 817 | 1.5286 |
| F | Phe | UUC | 357 | 0.6308 |
| F | Phe | UUU | 775 | 1.3692 |
| G | Gly | GGC | 155 | 0.448 |
| G | Gly | GGG | 247 | 0.714 |
| G | Gly | GGA | 551 | 1.5924 |
| G | Gly | GGU | 431 | 1.2456 |
| H | His | CAC | 110 | 0.4498 |
| H | His | CAU | 379 | 1.5502 |
| I | Ile | AUU | 858 | 1.5036 |
| I | Ile | AUA | 511 | 0.8955 |
| I | Ile | AUC | 343 | 0.6012 |
| K | Lys | AAA | 815 | 1.5106 |
| K | Lys | AAG | 264 | 0.4894 |
| L | Leu | CUA | 281 | 0.7992 |
| L | Leu | CUU | 447 | 1.2708 |
| L | Leu | UUG | 440 | 1.251 |
| L | Leu | CUG | 144 | 0.4092 |
| L | Leu | UUA | 674 | 1.9164 |
| L | Leu | CUC | 124 | 0.3528 |
| M | Met | AUG | 469 | 1 |
| N | Asn | AAC | 216 | 0.4444 |
| N | Asn | AAU | 756 | 1.5556 |
| P | Pro | CCC | 173 | 0.8132 |
| P | Pro | CCG | 123 | 0.578 |
| P | Pro | CCU | 315 | 1.4808 |
| P | Pro | CCA | 240 | 1.128 |
| Q | Gln | CAA | 574 | 1.5534 |
| Q | Gln | CAG | 165 | 0.4466 |
| R | Arg | CGA | 270 | 1.3548 |
| R | Arg | CGU | 265 | 1.3296 |
| R | Arg | AGG | 116 | 0.582 |
| R | Arg | CGG | 92 | 0.4614 |
| R | Arg | AGA | 358 | 1.7958 |
| R | Arg | CGC | 95 | 0.4764 |
| S | Ser | AGU | 323 | 1.2552 |
| S | Ser | UCG | 148 | 0.5754 |
| S | Ser | UCA | 293 | 1.1388 |
| S | Ser | UCU | 443 | 1.7214 |
| S | Ser | UCC | 255 | 0.9912 |
| S | Ser | AGC | 82 | 0.3186 |
| T | Thr | ACC | 192 | 0.7672 |
| T | Thr | ACU | 409 | 1.6344 |
| T | Thr | ACG | 98 | 0.3916 |
| T | Thr | ACA | 302 | 1.2068 |
| V | Val | GUG | 146 | 0.5216 |
| V | Val | GUA | 433 | 1.5464 |
| V | Val | GUU | 413 | 1.4752 |
| V | Val | GUC | 128 | 0.4572 |
| W | Trp | UGG | 369 | 1 |
| Y | Tyr | UAU | 590 | 1.6344 |
| Y | Tyr | UAC | 132 | 0.3656 |

**Table S8**. Putative optimal codons in chloroplast genome of of four *Salvia* species.

| *Salvia guidongensis* | | | | | | |
| --- | --- | --- | --- | --- | --- | --- |
| AA | Codon | HEG-nu | HEG-rscu | LEG-nu | LEG-rscu | ΔRSCU |
| Ala | GCA* | 10 | 1.38 | 35 | 1.21 | 0.17 |
|  | GCC** | 9 | 1.24 | 24 | 0.83 | 0.41 |
|  | GCG | 0 | 0 | 15 | 0.52 | -0.52 |
|  | GCU | 10 | 1.38 | 42 | 1.45 | -0.07 |
|  |  |  |  |  |  |  |
| Arg | AGA | 19 | 1.61 | 73 | 2.07 | -0.46 |
|  | AGG | 8 | 0.68 | 34 | 0.96 | -0.28 |
|  | CGA*** | 21 | 1.77 | 39 | 1.1 | 0.67 |
|  | CGC | 4 | 0.34 | 14 | 0.4 | -0.06 |
|  | CGG | 7 | 0.59 | 20 | 0.57 | 0.02 |
|  | CGU* | 12 | 1.01 | 32 | 0.91 | 0.1 |
|  |  |  |  |  |  |  |
| Asn | AAC | 9 | 0.42 | 38 | 0.42 | 0 |
|  | AAU | 34 | 1.58 | 144 | 1.58 | 0 |
|  |  |  |  |  |  |  |
| Asp | GAC | 4 | 0.44 | 35 | 0.4 | 0.04 |
|  | GAU | 14 | 1.56 | 140 | 1.6 | -0.04 |
|  |  |  |  |  |  |  |
| Cys | UGC | 1 | 0.4 | 13 | 0.62 | -0.22 |
|  | UGU* | 4 | 1.6 | 29 | 1.38 | 0.22 |
|  |  |  |  |  |  |  |
| Gln | CAA | 19 | 1.46 | 84 | 1.4 | 0.06 |
|  | CAG | 7 | 0.54 | 36 | 0.6 | -0.06 |
|  |  |  |  |  |  |  |
| Glu | GAA** | 28 | 1.65 | 105 | 1.23 | 0.42 |
|  | GAG | 6 | 0.35 | 66 | 0.77 | -0.42 |
|  |  |  |  |  |  |  |
| Gly | GGA** | 19 | 2.11 | 68 | 1.66 | 0.45 |
|  | GGC | 3 | 0.33 | 15 | 0.37 | -0.04 |
|  | GGG | 5 | 0.56 | 38 | 0.93 | -0.37 |
|  | GGU | 9 | 1 | 43 | 1.05 | -0.05 |
|  |  |  |  |  |  |  |
| His | CAC | 1 | 0.22 | 19 | 0.44 | -0.22 |
|  | CAU* | 8 | 1.78 | 68 | 1.56 | 0.22 |
|  |  |  |  |  |  |  |
| Ile | AUA | 17 | 0.85 | 77 | 0.93 | -0.08 |
|  | AUC | 6 | 0.3 | 64 | 0.77 | -0.47 |
|  | AUU*** | 37 | 1.85 | 108 | 1.3 | 0.55 |
|  |  |  |  |  |  |  |
| Leu | CUA | 6 | 0.53 | 46 | 0.87 | -0.34 |
|  | CUC | 3 | 0.26 | 28 | 0.53 | -0.27 |
|  | CUG | 5 | 0.44 | 29 | 0.55 | -0.11 |
|  | CUU | 9 | 0.79 | 70 | 1.33 | -0.54 |
|  | UUA*** | 23 | 2.03 | 61 | 1.16 | 0.87 |
|  | UUG** | 22 | 1.94 | 82 | 1.56 | 0.38 |
|  |  |  |  |  |  |  |
| Lys | AAA** | 29 | 1.61 | 123 | 1.29 | 0.32 |
|  | AAG | 7 | 0.39 | 68 | 0.71 | -0.32 |
|  |  |  |  |  |  |  |
| Met | AUG | 16 | 1 | 63 | 1 | 0 |
|  |  |  |  |  |  |  |
| Phe | UUC | 4 | 0.32 | 83 | 0.99 | -0.67 |
|  | UUU*** | 21 | 1.68 | 85 | 1.01 | 0.67 |
|  |  |  |  |  |  |  |
| Pro | CCA* | 5 | 1.25 | 32 | 0.97 | 0.28 |
|  | CCC | 4 | 1 | 31 | 0.94 | 0.06 |
|  | CCG* | 4 | 1 | 26 | 0.79 | 0.21 |
|  | CCU | 3 | 0.75 | 43 | 1.3 | -0.55 |
|  |  |  |  |  |  |  |
| Ser | AGC* | 3 | 0.36 | 13 | 0.27 | 0.09 |
|  | AGU*** | 13 | 1.56 | 50 | 1.06 | 0.5 |
|  | UCA | 10 | 1.2 | 58 | 1.23 | -0.03 |
|  | UCC | 6 | 0.72 | 52 | 1.1 | -0.38 |
|  | UCG | 4 | 0.48 | 34 | 0.72 | -0.24 |
|  | UCU | 14 | 1.68 | 77 | 1.63 | 0.05 |
|  |  |  |  |  |  |  |
| Thr | ACA | 6 | 0.75 | 43 | 1.26 | -0.51 |
|  | ACC*** | 10 | 1.25 | 22 | 0.64 | 0.61 |
|  | ACG | 1 | 0.12 | 26 | 0.76 | -0.64 |
|  | ACU*** | 15 | 1.88 | 46 | 1.34 | 0.54 |
|  |  |  |  |  |  |  |
| Trp | UGG | 4 | 1 | 60 | 1 | 0 |
|  |  |  |  |  |  |  |
| Tyr | UAC | 3 | 0.43 | 27 | 0.45 | -0.02 |
|  | UAU | 11 | 1.57 | 92 | 1.55 | 0.02 |
|  |  |  |  |  |  |  |
| Val | GUA | 6 | 0.86 | 35 | 1.17 | -0.31 |
|  | GUC | 2 | 0.29 | 19 | 0.63 | -0.34 |
|  | GUG | 6 | 0.86 | 25 | 0.83 | 0.03 |
|  | GUU*** | 14 | 2 | 41 | 1.37 | 0.63 |
|  |  |  |  |  |  |  |
| #0: The value is not exists | | | | | | |
| #HEG:High expression gene;LEG:Low expression gene;nu:number | | | | | | |
| #*: ΔRSCU >= 0.08, **: ΔRSCU >= 0.3, ***: ΔRSCU >= 0.5 | | | | | | |
| #ΔRSCU >= 0.08 (20): GCA,GCC,CGA,CGU,UGU,GAA,GGA,CAU,AUU,UUA,UUG,AAA,UUU,CCA,CCG,AGC,AGU,ACC,ACU,GUU  #RSCU > 1 (32): AAA,AAU,ACA,ACC,ACU,AGA,AGU,AUU,CAA,CAU,CCA,CCU,CGA,CGU,CUU,GAA,GAU,GCA,GCC,GCU,GGA,  GGU,GUA,GUU,UAU,UCA,UCC,UCU,UGU,UUA,UUG,UUU  #Preferred codons (18): GCA,GCC,CGA,CGU,UGU,GAA,GGA,CAU,AUU,UUA,UUG,AAA,UUU,CCA,AGU,ACC,ACU,GUU | | | | | | |
| *Salvia glutinosa* | | | | | | |
| AA | Codon | HEG-nu | HEG-rscu | LEG-nu | LEG-rscu | ΔRSCU |
| Ala | GCA* | 10 | 1.38 | 35 | 1.21 | 0.17 |
|  | GCC* | 8 | 1.1 | 24 | 0.83 | 0.27 |
|  | GCG | 0 | 0 | 15 | 0.52 | -0.52 |
|  | GCU | 11 | 1.52 | 42 | 1.45 | 0.07 |
|  |  |  |  |  |  |  |
| Arg | AGA | 19 | 1.61 | 73 | 2.07 | -0.46 |
|  | AGG | 8 | 0.68 | 34 | 0.96 | -0.28 |
|  | CGA*** | 22 | 1.86 | 38 | 1.08 | 0.78 |
|  | CGC | 4 | 0.34 | 15 | 0.42 | -0.08 |
|  | CGG | 6 | 0.51 | 20 | 0.57 | -0.06 |
|  | CGU* | 12 | 1.01 | 32 | 0.91 | 0.1 |
|  |  |  |  |  |  |  |
| Asn | AAC | 9 | 0.43 | 38 | 0.42 | 0.01 |
|  | AAU | 33 | 1.57 | 144 | 1.58 | -0.01 |
|  |  |  |  |  |  |  |
| Asp | GAC | 3 | 0.33 | 35 | 0.4 | -0.07 |
|  | GAU | 15 | 1.67 | 141 | 1.6 | 0.07 |
|  |  |  |  |  |  |  |
| Cys | UGC | 1 | 0.4 | 13 | 0.62 | -0.22 |
|  | UGU* | 4 | 1.6 | 29 | 1.38 | 0.22 |
|  |  |  |  |  |  |  |
| Gln | CAA | 18 | 1.44 | 84 | 1.4 | 0.04 |
|  | CAG | 7 | 0.56 | 36 | 0.6 | -0.04 |
|  |  |  |  |  |  |  |
| Glu | GAA** | 28 | 1.65 | 106 | 1.23 | 0.42 |
|  | GAG | 6 | 0.35 | 66 | 0.77 | -0.42 |
|  |  |  |  |  |  |  |
| Gly | GGA** | 19 | 2.11 | 68 | 1.66 | 0.45 |
|  | GGC | 3 | 0.33 | 15 | 0.37 | -0.04 |
|  | GGG | 5 | 0.56 | 38 | 0.93 | -0.37 |
|  | GGU | 9 | 1 | 43 | 1.05 | -0.05 |
|  |  |  |  |  |  |  |
| His | CAC | 1 | 0.22 | 19 | 0.43 | -0.21 |
|  | CAU* | 8 | 1.78 | 69 | 1.57 | 0.21 |
|  |  |  |  |  |  |  |
| Ile | AUA | 17 | 0.85 | 77 | 0.93 | -0.08 |
|  | AUC | 6 | 0.3 | 64 | 0.77 | -0.47 |
|  | AUU*** | 37 | 1.85 | 108 | 1.3 | 0.55 |
|  |  |  |  |  |  |  |
| Leu | CUA | 6 | 0.53 | 46 | 0.87 | -0.34 |
|  | CUC | 3 | 0.26 | 28 | 0.53 | -0.27 |
|  | CUG | 5 | 0.44 | 29 | 0.55 | -0.11 |
|  | CUU | 9 | 0.79 | 72 | 1.35 | -0.56 |
|  | UUA*** | 23 | 2.03 | 60 | 1.13 | 0.9 |
|  | UUG** | 22 | 1.94 | 84 | 1.58 | 0.36 |
|  |  |  |  |  |  |  |
| Lys | AAA** | 30 | 1.62 | 123 | 1.28 | 0.34 |
|  | AAG | 7 | 0.38 | 69 | 0.72 | -0.34 |
|  |  |  |  |  |  |  |
| Met | AUG | 16 | 1 | 63 | 1 | 0 |
|  |  |  |  |  |  |  |
| Phe | UUC | 4 | 0.32 | 85 | 1 | -0.68 |
|  | UUU*** | 21 | 1.68 | 85 | 1 | 0.68 |
|  |  |  |  |  |  |  |
| Pro | CCA* | 5 | 1.25 | 33 | 0.99 | 0.26 |
|  | CCC | 4 | 1 | 31 | 0.93 | 0.07 |
|  | CCG* | 4 | 1 | 26 | 0.78 | 0.22 |
|  | CCU | 3 | 0.75 | 43 | 1.29 | -0.54 |
|  |  |  |  |  |  |  |
| Ser | AGC* | 3 | 0.35 | 13 | 0.27 | 0.08 |
|  | AGU*** | 14 | 1.65 | 50 | 1.05 | 0.6 |
|  | UCA | 10 | 1.18 | 58 | 1.22 | -0.04 |
|  | UCC | 6 | 0.71 | 53 | 1.12 | -0.41 |
|  | UCG | 4 | 0.47 | 34 | 0.72 | -0.25 |
|  | UCU | 14 | 1.65 | 77 | 1.62 | 0.03 |
|  |  |  |  |  |  |  |
| Thr | ACA | 6 | 0.75 | 43 | 1.26 | -0.51 |
|  | ACC*** | 10 | 1.25 | 22 | 0.64 | 0.61 |
|  | ACG | 1 | 0.12 | 26 | 0.76 | -0.64 |
|  | ACU*** | 15 | 1.88 | 46 | 1.34 | 0.54 |
|  |  |  |  |  |  |  |
| Trp | UGG | 4 | 1 | 60 | 1 | 0 |
|  |  |  |  |  |  |  |
| Tyr | UAC | 3 | 0.43 | 27 | 0.46 | -0.03 |
|  | UAU | 11 | 1.57 | 91 | 1.54 | 0.03 |
|  |  |  |  |  |  |  |
| Val | GUA | 6 | 0.86 | 35 | 1.17 | -0.31 |
|  | GUC | 2 | 0.29 | 19 | 0.63 | -0.34 |
|  | GUG | 6 | 0.86 | 25 | 0.83 | 0.03 |
|  | GUU*** | 14 | 2 | 41 | 1.37 | 0.63 |
| #ΔRSCU >= 0.08 (20): GCA,GCC,CGA,CGU,UGU,GAA,GGA,CAU,AUU,UUA,UUG,AAA,UUU,CCA,CCG,AGC,AGU,ACC,ACU,GUU | | | | | | |
| #RSCU > 1 (32): AAA,AAU,ACA,ACC,ACU,AGA,AGU,AUU,CAA,CAU,CCA,CCU,CGA,CGU,CUU,GAA,GAU,GCA,GCC,GCU,GGA,GGU,GUA,GUU,UAU,UCA,UCC,UCU,UGU,UUA,UUG,UUU | | | | | | |
| #Preferred codons (18): GCA,GCC,CGA,CGU,UGU,GAA,GGA,CAU,AUU,UUA,UUG,AAA,UUU,CCA,AGU,ACC,ACU,GUU | | | | | | |
|  |  |  | *Salvia nubicola* | |  |  |
| AA | Codon | HEG-nu | HEG-rscu | LEG-nu | LEG-rscu | ΔRSCU |
| Ala | GCA* | 10 | 1.38 | 35 | 1.21 | 0.17 |
|  | GCC** | 9 | 1.24 | 24 | 0.83 | 0.41 |
|  | GCG | 0 | 0 | 15 | 0.52 | -0.52 |
|  | GCU | 10 | 1.38 | 42 | 1.45 | -0.07 |
|  |  |  |  |  |  |  |
| Arg | AGA | 19 | 1.61 | 72 | 2.05 | -0.44 |
|  | AGG | 8 | 0.68 | 34 | 0.97 | -0.29 |
|  | CGA*** | 22 | 1.86 | 38 | 1.08 | 0.78 |
|  | CGC | 4 | 0.34 | 15 | 0.43 | -0.09 |
|  | CGG | 7 | 0.59 | 20 | 0.57 | 0.02 |
|  | CGU | 11 | 0.93 | 32 | 0.91 | 0.02 |
|  |  |  |  |  |  |  |
| Asn | AAC | 9 | 0.43 | 38 | 0.42 | 0.01 |
|  | AAU | 33 | 1.57 | 144 | 1.58 | -0.01 |
|  |  |  |  |  |  |  |
| Asp | GAC | 4 | 0.44 | 35 | 0.4 | 0.04 |
|  | GAU | 14 | 1.56 | 141 | 1.6 | -0.04 |
|  |  |  |  |  |  |  |
| Cys | UGC | 1 | 0.33 | 13 | 0.62 | -0.29 |
|  | UGU* | 5 | 1.67 | 29 | 1.38 | 0.29 |
|  |  |  |  |  |  |  |
| Gln | CAA* | 18 | 1.5 | 84 | 1.4 | 0.1 |
|  | CAG | 6 | 0.5 | 36 | 0.6 | -0.1 |
|  |  |  |  |  |  |  |
| Glu | GAA** | 28 | 1.65 | 105 | 1.22 | 0.43 |
|  | GAG | 6 | 0.35 | 67 | 0.78 | -0.43 |
|  |  |  |  |  |  |  |
| Gly | GGA** | 19 | 2.11 | 69 | 1.67 | 0.44 |
|  | GGC | 3 | 0.33 | 15 | 0.36 | -0.03 |
|  | GGG | 5 | 0.56 | 38 | 0.92 | -0.36 |
|  | GGU | 9 | 1 | 43 | 1.04 | -0.04 |
|  |  |  |  |  |  |  |
| His | CAC | 1 | 0.22 | 19 | 0.43 | -0.21 |
|  | CAU* | 8 | 1.78 | 69 | 1.57 | 0.21 |
|  |  |  |  |  |  |  |
| Ile | AUA | 17 | 0.85 | 76 | 0.92 | -0.07 |
|  | AUC | 6 | 0.3 | 64 | 0.77 | -0.47 |
|  | AUU*** | 37 | 1.85 | 108 | 1.31 | 0.54 |
|  |  |  |  |  |  |  |
| Leu | CUA | 6 | 0.53 | 47 | 0.88 | -0.35 |
|  | CUC | 3 | 0.26 | 28 | 0.53 | -0.27 |
|  | CUG | 5 | 0.44 | 29 | 0.54 | -0.1 |
|  | CUU | 9 | 0.79 | 72 | 1.35 | -0.56 |
|  | UUA*** | 23 | 2.03 | 60 | 1.12 | 0.91 |
|  | UUG** | 22 | 1.94 | 84 | 1.57 | 0.37 |
|  |  |  |  |  |  |  |
| Lys | AAA** | 30 | 1.62 | 123 | 1.28 | 0.34 |
|  | AAG | 7 | 0.38 | 69 | 0.72 | -0.34 |
|  |  |  |  |  |  |  |
| Met | AUG | 16 | 1 | 63 | 1 | 0 |
|  |  |  |  |  |  |  |
| Phe | UUC | 4 | 0.32 | 85 | 1 | -0.68 |
|  | UUU*** | 21 | 1.68 | 85 | 1 | 0.68 |
|  |  |  |  |  |  |  |
| Pro | CCA* | 5 | 1.25 | 33 | 0.99 | 0.26 |
|  | CCC | 4 | 1 | 31 | 0.93 | 0.07 |
|  | CCG* | 4 | 1 | 26 | 0.78 | 0.22 |
|  | CCU | 3 | 0.75 | 43 | 1.29 | -0.54 |
|  |  |  |  |  |  |  |
| Ser | AGC* | 3 | 0.35 | 13 | 0.27 | 0.08 |
|  | AGU*** | 14 | 1.65 | 50 | 1.05 | 0.6 |
|  | UCA | 10 | 1.18 | 58 | 1.22 | -0.04 |
|  | UCC | 6 | 0.71 | 52 | 1.09 | -0.38 |
|  | UCG | 4 | 0.47 | 34 | 0.72 | -0.25 |
|  | UCU | 14 | 1.65 | 78 | 1.64 | 0.01 |
|  |  |  |  |  |  |  |
| Thr | ACA | 6 | 0.75 | 43 | 1.26 | -0.51 |
|  | ACC*** | 10 | 1.25 | 22 | 0.64 | 0.61 |
|  | ACG | 1 | 0.12 | 26 | 0.76 | -0.64 |
|  | ACU*** | 15 | 1.88 | 46 | 1.34 | 0.54 |
|  |  |  |  |  |  |  |
| Trp | UGG | 4 | 1 | 60 | 1 | 0 |
|  |  |  |  |  |  |  |
| Tyr | UAC | 3 | 0.43 | 27 | 0.46 | -0.03 |
|  | UAU | 11 | 1.57 | 91 | 1.54 | 0.03 |
|  |  |  |  |  |  |  |
| Val | GUA | 6 | 0.86 | 35 | 1.17 | -0.31 |
|  | GUC | 2 | 0.29 | 19 | 0.63 | -0.34 |
|  | GUG | 6 | 0.86 | 25 | 0.83 | 0.03 |
|  | GUU*** | 14 | 2 | 41 | 1.37 | 0.63 |
| #ΔRSCU>=0.08(20): GCA,GCC,CGA,UGU,CAA,GAA,GGA,CAU,AUU,UUA,UUG,AAA,UUU,CCA,CCG,AGC,AGU,ACC,ACU,GUU | | | | | | |
| #RSCU>1(31):AAA,AAU,ACA,ACC,ACU,AGA,AGU,AUU,CAA,CAU,CCA,CCU,CGA,CUU,GAA,GAU,GCA,GCC,GCU,GGA,GGU,GUA,GUU,UAU,UCA,UCC,UCU,UGU,UUA,UUG,UUU | | | | | | |
| #Preferred codons(18): GCA,GCC,CGA,UGU,CAA,GAA,GGA,CAU,AUU,UUA,UUG,AAA,UUU,CCA,AGU,ACC,ACU,GUU | | | | | | |
| *Salvia chanryoenica* | | | | | | |
| AA | Codon | HEG-nu | HEG-rscu | LEG-nu | LEG-rscu | ΔRSCU |
| Ala | GCA*** | 13 | 1.68 | 34 | 1.18 | 0.5 |
|  | GCC | 4 | 0.52 | 24 | 0.83 | -0.31 |
|  | GCG | 3 | 0.39 | 15 | 0.52 | -0.13 |
|  | GCU | 11 | 1.42 | 42 | 1.46 | -0.04 |
|  |  |  |  |  |  |  |
| Arg | AGA | 20 | 1.94 | 73 | 2.07 | -0.13 |
|  | AGG | 6 | 0.58 | 34 | 0.96 | -0.38 |
|  | CGA*** | 17 | 1.65 | 38 | 1.08 | 0.57 |
|  | CGC | 4 | 0.39 | 15 | 0.42 | -0.03 |
|  | CGG | 3 | 0.29 | 20 | 0.57 | -0.28 |
|  | CGU* | 12 | 1.16 | 32 | 0.91 | 0.25 |
|  |  |  |  |  |  |  |
| Asn | AAC* | 9 | 0.53 | 38 | 0.42 | 0.11 |
|  | AAU | 25 | 1.47 | 144 | 1.58 | -0.11 |
|  |  |  |  |  |  |  |
| Asp | GAC* | 4 | 0.47 | 34 | 0.39 | 0.08 |
|  | GAU | 13 | 1.53 | 141 | 1.61 | -0.08 |
|  |  |  |  |  |  |  |
| Cys | UGC | 1 | 0.4 | 13 | 0.62 | -0.22 |
|  | UGU* | 4 | 1.6 | 29 | 1.38 | 0.22 |
|  |  |  |  |  |  |  |
| Gln | CAA** | 13 | 1.73 | 85 | 1.41 | 0.32 |
|  | CAG | 2 | 0.27 | 36 | 0.59 | -0.32 |
|  |  |  |  |  |  |  |
| Glu | GAA** | 21 | 1.62 | 105 | 1.23 | 0.39 |
|  | GAG | 5 | 0.38 | 66 | 0.77 | -0.39 |
|  |  |  |  |  |  |  |
| Gly | GGA | 15 | 1.71 | 68 | 1.66 | 0.05 |
|  | GGC | 3 | 0.34 | 15 | 0.37 | -0.03 |
|  | GGG | 1 | 0.11 | 38 | 0.93 | -0.82 |
|  | GGU*** | 16 | 1.83 | 43 | 1.05 | 0.78 |
|  |  |  |  |  |  |  |
| His | CAC | 1 | 0.22 | 19 | 0.44 | -0.22 |
|  | CAU* | 8 | 1.78 | 67 | 1.56 | 0.22 |
|  |  |  |  |  |  |  |
| Ile | AUA | 16 | 0.87 | 77 | 0.93 | -0.06 |
|  | AUC | 5 | 0.27 | 64 | 0.77 | -0.5 |
|  | AUU*** | 34 | 1.85 | 108 | 1.3 | 0.55 |
|  |  |  |  |  |  |  |
| Leu | CUA | 7 | 0.59 | 46 | 0.87 | -0.28 |
|  | CUC | 4 | 0.34 | 28 | 0.53 | -0.19 |
|  | CUG | 2 | 0.17 | 29 | 0.55 | -0.38 |
|  | CUU | 13 | 1.1 | 72 | 1.35 | -0.25 |
|  | UUA*** | 25 | 2.11 | 61 | 1.15 | 0.96 |
|  | UUG* | 20 | 1.69 | 83 | 1.56 | 0.13 |
|  |  |  |  |  |  |  |
| Lys | AAA** | 30 | 1.71 | 122 | 1.28 | 0.43 |
|  | AAG | 5 | 0.29 | 69 | 0.72 | -0.43 |
|  |  |  |  |  |  |  |
| Met | AUG | 16 | 1 | 63 | 1 | 0 |
|  |  |  |  |  |  |  |
| Phe | UUC | 3 | 0.22 | 84 | 0.99 | -0.77 |
|  | UUU*** | 24 | 1.78 | 85 | 1.01 | 0.77 |
|  |  |  |  |  |  |  |
| Pro | CCA* | 9 | 1.16 | 33 | 0.99 | 0.17 |
|  | CCC | 7 | 0.9 | 31 | 0.93 | -0.03 |
|  | CCG | 6 | 0.77 | 26 | 0.78 | -0.01 |
|  | CCU | 9 | 1.16 | 43 | 1.29 | -0.13 |
|  |  |  |  |  |  |  |
| Ser | AGC | 2 | 0.28 | 13 | 0.27 | 0.01 |
|  | AGU** | 10 | 1.4 | 50 | 1.05 | 0.35 |
|  | UCA* | 10 | 1.4 | 58 | 1.22 | 0.18 |
|  | UCC | 7 | 0.98 | 53 | 1.12 | -0.14 |
|  | UCG | 4 | 0.56 | 34 | 0.72 | -0.16 |
|  | UCU | 10 | 1.4 | 77 | 1.62 | -0.22 |
|  |  |  |  |  |  |  |
| Thr | ACA | 10 | 1.29 | 43 | 1.26 | 0.03 |
|  | ACC*** | 9 | 1.16 | 22 | 0.64 | 0.52 |
|  | ACG | 0 | 0 | 26 | 0.76 | -0.76 |
|  | ACU* | 12 | 1.55 | 46 | 1.34 | 0.21 |
|  |  |  |  |  |  |  |
| Trp | UGG | 6 | 1 | 60 | 1 | 0 |
|  |  |  |  |  |  |  |
| Tyr | UAC | 1 | 0.14 | 27 | 0.45 | -0.31 |
|  | UAU** | 13 | 1.86 | 92 | 1.55 | 0.31 |
|  |  |  |  |  |  |  |
| Val | GUA* | 11 | 1.33 | 36 | 1.19 | 0.14 |
|  | GUC | 2 | 0.24 | 19 | 0.63 | -0.39 |
|  | GUG | 3 | 0.36 | 25 | 0.83 | -0.47 |
|  | GUU*** | 17 | 2.06 | 41 | 1.36 | 0.7 |
| #ΔRSCU >= 0.08 (23): GCA,CGA,CGU,AAC,GAC,UGU,CAA,GAA,GGU,CAU,AUU,UUA,UUG,AAA,UUU,CCA,AGU,UCA,ACC,ACU,UAU,GUA,GUU | | | | | | |
| #RSCU > 1 (31): AAA,AAU,ACA,ACC,ACU,AGA,AGU,AUU,CAA,CAU,CCA,CCU,CGA,CGU,CUU,GAA,GAU,GCA,GCU,GGA,GGU,GUA,GUU,UAU,UCA,UCC,UCU,UGU,UUA,UUG,UUU | | | | | | |
| #Preferred codons (21): GCA,CGA,CGU,UGU,CAA,GAA,GGU,CAU,AUU,UUA,UUG,AAA,UUU,CCA,AGU,UCA,ACC,ACU,UAU,GUA,GUU | | | | | | |

**Table S9** Correlation analysis of parameters of chloroplast genomic codon of four *Salvia* species.

| *Salvia guidongensis* | | | | | | |
| --- | --- | --- | --- | --- | --- | --- |
| Variation | GC1 | GC2 | GC3 | GC_all | ENC | condon.No. |
| GC1 | 1 | 0.474** | 0.216 | 0.848** | 0.239 | -0.138 |
| GC2 | 0.474** | 1 | 0.078 | 0.767** | -0.048 | -0.234 |
| GC3 | 0.216 | 0.078 | 1 | 0.494** | 0.455** | 0.309* |
| GC_all | 0.848** | 0.767** | 0.494** | 1 | 0.261 | -0.083 |
| ENC | 0.239 | -0.048 | 0.455** | 0.261 | 1 | 0.287* |
| codon.No. | -0.138 | -0.234 | 0.309* | -0.083 | 0.287* | 1 |
| *Salvia glutinosa* | | | | | | |
| Variation | GC1 | GC2 | GC3 | GC_all | ENC | condon.No. |
| GC1 | 1 | 0.472** | 0.23 | 0.847** | 0.235 | -0.145 |
| GC2 | 0.472** | 1 | 0.096 | 0.764** | -0.029 | -0.239 |
| GC3 | 0.23 | 0.096 | 1 | 0.515** | 0.482** | 0.330* |
| GC_all | 0.847** | 0.764** | 0.515** | 1 | 0.280* | -0.079 |
| ENC | 0.235 | -0.029 | 0.482** | 0.280* | 1 | 0.289* |
| codon.No. | -0.145 | -0.239 | 0.330* | -0.079 | 0.289* | 1 |
| *Salvia nubicola* | | | | | | |
| Variation | GC1 | GC2 | GC3 | GC_all | ENC | condon.No. |
| GC1 | 1 | 0.478** | 0.222 | 0.847** | 0.234 | -0.134 |
| GC2 | 0.478** | 1 | 0.105 | 0.768** | -0.027 | -0.24 |
| GC3 | 0.222 | 0.105 | 1 | 0.513** | 0.458** | 0.315* |
| GC_all | 0.847** | 0.768** | 0.513** | 1 | 0.272 | -0.078 |
| ENC | 0.234 | -0.027 | 0.458** | 0.272 | 1 | 0.292* |
| codon.No. | -0.134 | -0.24 | 0.315* | -0.078 | 0.292* | 1 |
| *Salvia chanryoenica* | | | | | | |
| Variation | GC1 | GC2 | GC3 | GC_all | ENC | condon.No. |
| GC1 | 1 | 0.480** | 0.222 | 0.850** | 0.252 | -0.141 |
| GC2 | 0.480** | 1 | 0.093 | 0.769** | -0.012 | -0.231 |
| GC3 | 0.222 | 0.093 | 1 | 0.503** | 0.473** | 0.330* |
| GC_all | 0.850** | 0.769** | 0.503** | 1 | 0.292* | -0.075 |
| ENC | 0.252 | -0.012 | 0.473** | 0.292* | 1 | 0.297* |
| codon.No. | -0.141 | -0.231 | 0.330* | -0.075 | 0.297* | 1 |

* indicated significant correlation(P < 0.5); **indicated extremely significant correlation(P < 0.01)

**Table S10** Distribution of ENC ratio of four *Salvia* species.

| *Salvia guidongensis* | | | |
| --- | --- | --- | --- |
| Class range | Class mid value | Frequency number | Frequency |
| -0.05–0.05 | 0 | 7 | 0.13 |
| 0.05–0.15 | 0.1 | 35 | 0.67 |
| 0.15–0.25 | 0.2 | 7 | 0.13 |
| 0.25–0.35 | 0.3 | 3 | 0.06 |
| Tatal |  | 52 | 1 |
| *Salvia glutinosa* | | | |
| Class range | Class mid value | Frequency number | Frequency |
| -0.05–0.05 | 0 | 9 | 0.17 |
| 0.05–0.15 | 0.1 | 34 | 0.65 |
| 0.15–0.25 | 0.2 | 6 | 0.12 |
| 0.25–0.35 | 0.3 | 3 | 0.06 |
| Tatal |  | 52 | 1 |
| *Salvia nubicola* | | | |
| Class range | Class mid value | Frequency number | Frequency |
| -0.05–0.05 | 0 | 8 | 0.15 |
| 0.05–0.15 | 0.1 | 34 | 0.65 |
| 0.15–0.25 | 0.2 | 7 | 0.13 |
| 0.25–0.35 | 0.3 | 3 | 0.06 |
| Tatal |  | 52 | 1 |
| *Salvia chanryoenica* | | | |
| Class range | Class mid value | Frequency number | Frequency |
| -0.05–0.05 | 0 | 8 | 0.16 |
| 0.05–0.15 | 0.1 | 33 | 0.65 |
| 0.15–0.25 | 0.2 | 7 | 0.14 |
| 0.25–0.35 | 0.3 | 3 | 0.06 |
| Tatal |  | 51 | 1 |

**References:**

Bendiksby, M., Thorbek, L., Scheen, A., Lindqvist, C., and Ryding, O. (2011). An updated phylogeny and classification of Lamiaceae subfamily Lamioideae. *Taxon* 60, 471–484. doi:10.1002/tax.602015

Hu, G., Takano, A., Drew, B.T., Liu, E., and Soltis, D.E., et al. (2018). Phylogeny and staminal evolution of *Salvia* (Lamiaceae, Nepetoideae) in East Asia. *Annals of Botany* 122, 649–668. doi:10.1093/aob/mcy104/5045153

Scarcelli, N., Barnaud, A., Eiserhardt, W., Treier, U.A., and Seveno, M., et al. (2011). A set of 100 chloroplast DNA primer pairs to study population genetics and phylogeny in monocotyledons. *Plos One* 6, e19954. doi:10.1371/journal.pone.0019954

Shaw, J., Lickey, E.B., Schilling, E.E., and Small, R.L. (2007). Comparison of whole chloroplast genome sequences to choose noncoding regions for phylogenetic studies in angiosperms: the tortoise and the hare III. *American Journal of Botany* 94, 275–288. doi:10.3732/ajb.94.3.275
